# Oligonucleotide Array Comparative Genomic Hybridization (oaCGH) based characterization of genetic deficiencies as an aid to gene mapping in *Caenorhabditis elegans*

**DOI:** 10.1186/1471-2164-8-402

**Published:** 2007-11-07

**Authors:** Martin R Jones, Jason S Maydan, Stephane Flibotte, Donald G Moerman, David L Baillie

**Affiliations:** 1 Department of Molecular Biology and Biochemistry, Simon Fraser University, Burnaby BC V35 1S6 Canada; 2 Canada's Michael Smith Genome Sciences Centre, BC Cancer Agency, Vancouver, British Columbia V5Z 4S6 Canada; 3 Department of Zoology, University of British Columbia, Vancouver, British Columbia V6T 1Z4, Canada

## Abstract

**Background:**

A collection of genetic deficiencies covering over 70% of the *Caenorhabditis elegans *genome exists, however the application of these valuable biological tools has been limited due to the incomplete correlation between their genetic and physical characterization.

**Results:**

We have applied oligonucleotide array Comparative Genomic Hybridization (oaCGH) to the high resolution, molecular characterization of several genetic deficiency and duplication strains in a 5 Mb region of Chromosome III. We incorporate this data into a physical deficiency map which is subsequently used to direct the positional cloning of essential genes within the region. From this analysis we are able to quickly determine the molecular identity of several previously unidentified mutations.

**Conclusion:**

We have applied accurate, high resolution molecular analysis to the characterization of genetic mapping tools in *Caenorhabditis elegans*. Consequently we have generated a valuable physical mapping resource, which we have demonstrated can aid in the rapid molecular identification of mutations of interest.

## Background

A large resource of deletion strains (also known as genetic deficiencies) accounting for over 70% of the *Caenorhabditis elegans *genome has been generated by various research groups over the past three decades [1]. These genetic deficiencies have proven advantageous for a variety of purposes including; characterization of mutant alleles [2], identification of specific loci affecting developmental processes [3], investigation of genome replication and stability [4,5] and, most significantly, as tools for positional cloning of unmapped mutations to discrete regions of the genome [1,6-8].

The full potential of these biological tools has however been limited due to the lack of high resolution characterization at a genome wide scale. The mapping of physical breakpoint positions within each deficiency strain is required to allow the deleted gene complement for that strain to be precisely defined. Additionally, genetic deficiencies may exhibit molecular complexity preventing their reliable use in mapping experiments [9].

Previously, characterization of genetic deficiencies has been performed by fairly low resolution or labor intensive techniques such as genetic linkage mapping, PCR analysis [7] and, more recently, by the application of snip-SNP [9,10] and as a consequence many available deficiency strains remain poorly characterized.

Oligonucleotide array Comparative Genomic Analysis (oaCGH) is an emerging technology for high resolution mapping of chromosomal copy number changes at a genome wide scale through the comparison of the DNA ratio between two samples from the same organism [11,12]. The recent development of a *C. elegans *specific oaCGH platform for identification of novel single gene deletions [13] represents a powerful technology that can be adapted to the rapid and precise characterization of deficiency mapping strains.

In this study we demonstrate the successful application of oaCGH to the physical characterization of deficiency strains in *C. elegans*. We use this data to annotate a physical deficiency map within a 5 Mb region of chromosome III and demonstrate the application of this map to aid in the molecular identification of previously generated mutations known to reside within this region.

## Results and Discussion

### oaCGH mapped deletions and duplications physically define 17 zones around the dpy-17 region of Chromosome III

7 deficiencies and 2 duplications lying in the region of *dpy-17 *on chromosome III were chosen for oaCGH analysis (Nimblegen) as they have been previously characterized by both genetic linkage and PCR analysis, and used to roughly position a large number of unidentified EMS generated lethal mutants [7]. After oaCGH mapping had precisely defined the gene complement for each of these deficiencies a refined candidate gene approach was implemented to rapidly identify mutations in essential genes which map to this region.

Available mapping data for deficiency strains positioned deletion and duplication breakpoints with an average resolution of 117 kb. With the incorporation of the oaCGH data however, breakpoints resolved to within an average of 5.6 kb and subsequent analysis through PCR amplification and sequencing allowed for the identification of precise physical breakpoints in strains containing *nDf20, sDf121*, *sDf125*, *sD128 *and *sDf135 *(Table 1). The remaining deficiency strains, *sDf127 *and *sDf130*, both contain breakpoints which reside in relatively large intergenic regions and since the oaCGH array used for these samples has low probe density in intergenic regions [13] PCR and sequencing analysis was not feasible in these cases.

**Table 1:** Summary of mapping resolution for deficiencies using both genetic and molecular mapping techniques.

				Genetic/PCR Mapping Data		oaCGH Mapping Data		^*a*^Sequenced Breakpoint Coordinates		
					
Strain	Deficiency	Mutagen	Position	LEFT Cosmid	RIGHT Cosmid	LEFT (bp)	RIGHT (bp)	LEFT (bp)	RIGHT (bp)	Size of deficiency (kb)
MT3022	*nDf20*	GRI	Out	*egl-5*	*sma-2*	7895240	8678216	7896734	8676211	780
			In	*lin-36*	*dpy-19*	7900966	8676164			
BC4698	*sDf121*	GRI	Out	F10G11	C05D2	4568979	5637318	4568042	5637312	1069
			In	F35G12	F54E7	4569058	5636068			
BC4634	*sDf125*	UVI	Out	F42A10	K04C2	6178564	6620842	6178994	6620106	441
			In	C23G10	R13F6	6181034	6619737			
BC4638	*sDf127*	UVI	Out	C23G10	R13A5	6200904	7453868	ND	ND	^*d*^1194–1253
			In	C23G10	T20B12	6200951	7395215			
BC4690	*sDf128*	GRI	Out	C29E4	C06G4	7945153	8023540	7945392	8023387	78
			In	C06G4	F44B9	7945526	8019512			
BC4637	*sDf130*	UV	Out	C32A3	C05D2	3640014	5566628	3640101	5565581	1925
			In			3640119	5565449			
			Out			5635618	5664335	ND	ND	^*e*^16–29
			In	R13G10	ZC155	5645065	5661035			
BC4677	*sDf135*	GRI	Out	B0361	T20B12	7276848	7479897	7277386	7476402	199
			In	T20B12	R13A5	7277471	7467958			
^*b*^NA	*sDp3*	GRI	Out	*daf-7*	*dpy-19*	^*c*^Complex	8611252	NA	NA	NA
			In	*dpy-18*	*mig-10*		8611166			
BC3737	*sDp8*	UVI	Out	*let-721*	*sma-2*	6559307	8714234	NA	NA	NA
			In	M01G4	*dpy-19*	6562088	8710120			

Average resolution (kb)				117		5.6		0	0	

^*a*^Breakpoint coordinates aligned to Wormbase (release WS170).

^*b*^*sDp3 *structure is inferred from the background of the oaCGH data.

^*c*^The left breakpoint of *sDp3 *is fragmented over a large region.

^*d*^The right breakpoint of *sDf127 *falls within a 50 kb region of low complexity and therefore low probe density and could not be accurately defined.

^*e*^The second deleted region in *sDf130 *was not be resolved by PCR analysis.

**Figure 1:**
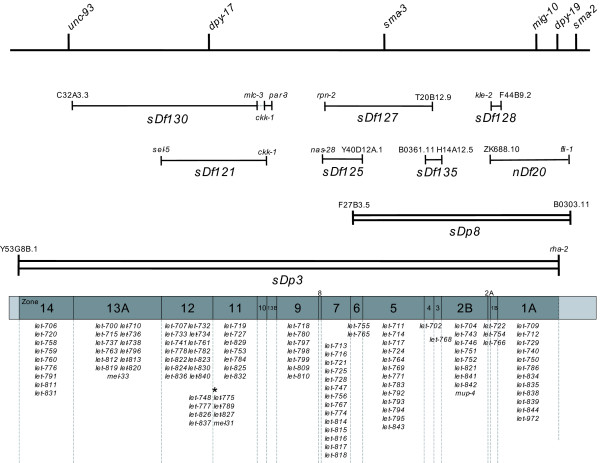
**A revised genetic and physical map of the *dpy-17 *region of chromosome III after incorporation of oaCGH deficiency mapping data**. Deficiencies are represented as single lines and duplications as double lines. The outermost genes residing within each deficiency are shown. 17 zones defined by the deficiencies and duplications are represented by grey boxes. Note that the transition between zones 11 and 12 is defined by the position of *dpy-17*. *8 lethal mutants have not been positioned left/right with respect to *dpy-17*. Figure is not to scale.

By using the refined mapping data obtained from the oaCGH analysis we annotated the deleted gene complement within each mapping strain, creating a physical map of zones defined by the overlap of the deficiencies and duplications within the region. The resulting map extends across almost 5 Mb of the genome and contains 17 zones with an average size of 323 kb (Figure 1). Zone 13 is the largest region, covering 929 kb and containing 204 predicted genes, while zone 8 is the smallest at 22 kb and contains 5 predicted genes (Table 2). Finally, positional mapping data available for mutations known to fall within this region was incorporated into the map, and in this way we have been able to assign each mutation to an accurate and precisely defined list of gene candidates (Figure 1. and Additional file 1).

**Table 2:** Physical and genetic structure of zones 1A-14.

Zone	Start (bp)	End (bp)	Left Gene	Right Gene	Size (kb)	# genes	^*b*^# RNAi Lethal	# Lethal Mutants
1A	8023387	8611252	F44B9.2	*rha-2*	588	151	39	12
1B	7945392	8023387	*kle-2*	F44B9.2	78	23	6	3
2A	7896734	7945392	ZK688.2	*kle-2*	49	14	5	0
2B	7476402	7896734	H14A12.5	ZK688.2	420	81	23	11
3	7395219	7476402	T20B12.9	H14A12.5	81	8	5	1
4	7277442	7395219	B0361.11	T20B12.9	118	35	11	1
5	6620106	7277442	Y40D12A.1	B0361.11	657	154	48	13
6	6559307	6620106	F27B3.5	Y40D12A.1	61	14	4	2
7	6200904	6562088	*rpn-2*	F27B3.5	361	97	25	15
8	6178994	6200951	*nas-28*	*rpn-2*	22	5	3	0
9	5662800	6178564	*par-3*	*nas-28*	516	123	29	7
^*a*^13	5637312	5664335	*ckk-1*	*par-3*	27	5	1	0
10	5565581	5637312	*mlc-3*	*ckk-1*	72	18	2	0
11	5107800	5565581	*dpy-17*	*mlc-3*	458	98	31	7 ^*c*^(+/- 8)
12	4569043	5107800	F35G12.2	*dpy-17*	539	163	46	14 ^*c*^(+/- 8)
13	3640101	4569043	C32A3.3	F35G21.1	929	204	54	13
14	3115819	3640101	Y53G8B.1	C32A3.3	524	85	21	9

^*a*^Zones 11 and 12 are defined by *dpy-17*.

^*b *^RNAi Lethal encompasses all genes annotated in Wormbase (release WS170) as having either emb, let, lvl and/or ste phenotypes by RNAi.

^*c *^8 lethal mutations have not been position right/left with respect to *dpy-17*.

### The free translocation sDp3 undergoes significant modification within deficiency strains

**Figure 2:**
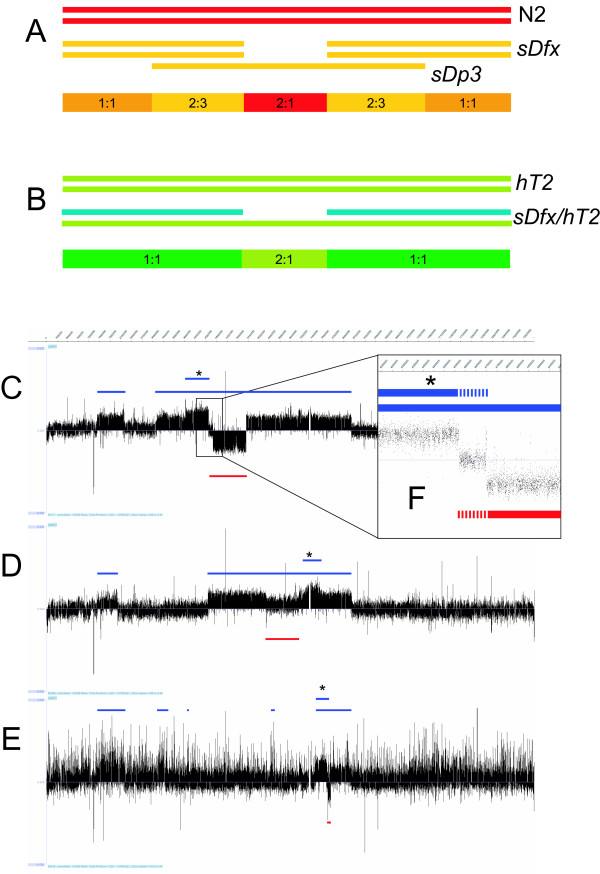
**oaCGH experimental overview and *sDp3 *balanced deficiency data**. (A-B) Schematic representation of the oaCGH experimental approaches; (A) using *sDp3 *balanced deficiency strains and (B) modified to use *hT2 (qIs48) *heterozygous balancer. Colored bars beneath schematic indicate expected DNA ratios. (C-E) oaCGH data obtained in GFF file format for three *sDp3 *balanced deficiency strains visualized using the SignalMap™ browser software [20]. (C) BC4697 (*sDf121 [sDp3])*, (D) BC4638 (*sDf127 [sDp3]) *and (E) BC4690 (*sDf128 [sDp3])*. Regions covered by *sDp3 *are represented as blue lines above the data; deletions are represented by red lines below the data. (F) Expansion of oaCGH data across the left breakpoint region of *sDf121*. Region of deletion ambiguity is represented by broken lines. *Apparent internal duplications of *sDp3*.

In our initial set of experiments we performed oaCGH using genomic DNA from three representative deficiency strains balanced by the free duplication *sDp3 *; BC4697 (*sDf121(s2098) [sDp3])*, BC4638 (*sDf127(s2428) [sDp3]) *and BC4690 (*sDf128(s2786) [sDp3]) *(Figure 2), using wild type Bristol N2 DNA as the reference sample. The expected ratio of reference DNA to test using this approach is 1:1 over the region of the chromosome not covered by *sDp3*, 2:3 over the region covered by *sDp3 *alone and 2:1 in the region of the deficiency balanced by *sDp3 *(Figure 2A).

This initial analysis revealed that the extent of *sDp3 *varies dramatically between each of the strains tested (Figure 2B–D). Since duplications maintained in balancer strains are known to breakdown both spontaneously and under the mutagenic conditions used to induce deficiencies [14-16] it is likely that the observed modifications to *sDp3 *are as a result of these factors.

In addition to the variability of *sDp3 *coverage, discrete duplications internal to *sDp3 *are observed within each of the strains tested (Figure 2). This lack of uniformity makes determination of deficiency structure problematic in some cases. For example, the presence of an internal duplication in BC4697 across the region containing the left breakpoint of *sDf121 *results in an increase of predicted copy number in this region (Figure 2F), while multiple variations within both *sDp3 *balanced strains containing *sDf127 *and *sDf128 *complicates the distinction between where DNA has been deleted or duplicated (Figure 2C–E and data not shown).

### oaCGH of heterozygous deficiencies resolves copy number ambiguity and results in precise mapping of deletion breakpoints

Since heterozygous deletions can be easily resolved with oaCGH [13] we surmised that the creation of single copy deficiency strains would eliminate the ambiguity introduced through the use of the *sDp3 *balancer. By using this approach the expected ratio of reference DNA to test is 1:1 along the whole length of the chromosome except within the deleted region where a ratio of 2:1 will be expected (Figure 2B).

To implement this strategy all of the *sDp3 *balanced deficiency strains to be tested were out-crossed to JK2689, which contains the GFP marked heterozygous balancer *hT2 (qIs48) *known to cover the region under investigation in this study [1]. For this analysis genomic JK2689 DNA was used as the reference sample to eliminate any genomic variability that may have been introduced into this strain through the heavy mutagenesis used for its original construction [17].

**Figure 3:**
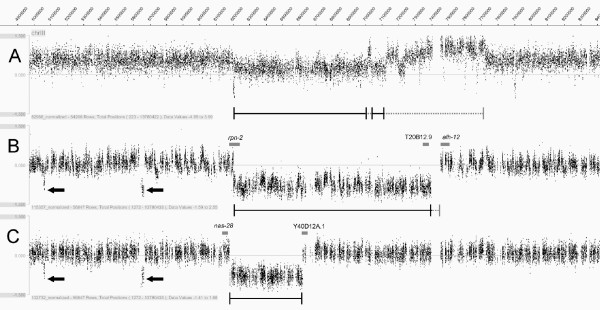
**Comparison of oaCGH data in the *sDp3 *and *hT2 (qIs48) *balancer backgrounds: **(A) BC4638 *(sDf127 [sDp3]) *(B) BC7163 *(sDf127/hT2 [qIs48]) *(C) BC7162 *(sDf125/hT2 [qIs48])*. Unambiguous deficiency mapping for each experiment is depicted as a solid black line while ambiguous regions are represented as a dashed grey line. Genes residing at the confirmed *sDf127 *breakpoints are shown (grey bars). Non-deficiency specific copy number changes introduced by the background strain can be seen (B and C solid arrows). Note: oaCGH array used in A is the Nimblegen design [20] while the array used in B and C is from the exon-centric array described by Mayden et.al [13] (see Methods).

With the elimination of *sDp3 *from the background the ability to accurately resolve breakpoint positions in the ambiguously mapped deficiency strains improved significantly. A comparison of oaCGH data for *sDf127 *balanced by *sDp3 *and this same deficiency out-crossed to *hT2 (qIs48) *highlights this improvement (Figure 3). As part of their construction, deficiency strains have been out-crossed several times and it is therefore unlikely that the process of creating the *hT2 *balanced strains has removed any features of these deficiencies. The oaCGH data obtained for each strain using the modified strategy is therefore likely to represent the initial structure of each of the deficiencies tested.

### Combining data from multiple array experiments allows for bias removal resulting in reliable deficiency characterization

oaCGH data obtained from separate strains using the same chip design performs consistently enough to allow for the integration and direct comparison of the data generated from multiple experiments. Through this data integration, common inconsistencies that are not specific to the deletion of interest are identified and eliminated from the annotation, resulting in a reliable characterization of the whole deficiency genome (Figure 3B and 3C). This is an important step given that small mutagenic events which may have occurred outside the known boundaries of a given deficiency may remain unidentified by traditional mapping methods and could lead to conflicting mapping data.

### oaCGH analysis reveals deficiency complexity on a genome-wide scale

We have analysed a number of deficiencies generated with both GRI and UV irradiation (Table 1 and data not shown), and have been unable to detect any significant mutagen specific deletion characteristics, the majority having a simple contiguous structure (Figure 3B and 3C, and data not shown). Exceptions to this observation are seen in the deficiencies *sDf130*, which contains two distinct deletions, the smaller of which defines zone 10 (Figure 1), and *sDf128 *which exhibits associated complexity.

**Figure 4:**
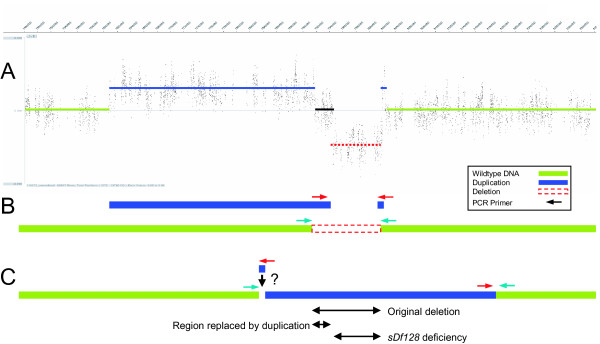
***sDf128 *associated complexity**. (A) BC7164 *(sDf128/hT2 [qIs48]) *oaCGH data showing retention of duplications. (B) Schematic representation of *sDf128 *PCR analysis and confirmed duplication insertion. (C) Predicted insertion of the duplicated region into the *sDf127 *genome. (?) Unconfirmed deficiency structure.

In a similar case as to that seen with *sDp3 *balanced *sDf121*, several duplications of various sizes extend into the deficiency region of *sDf128 *(Figure 2C and 2E). Unlike *sDf121 *however, these duplications are retained when *sDf128 *is out-crossed to *hT2 *(Figure 4), suggesting that they have been integrated into the deficiency genome. Attempts to further characterize the complexity of *sDf128 *through PCR amplification across the predicted deletion breakpoints failed, suggesting that integration of a duplicated region has occurred within the deficiency itself. Subsequent amplification of a PCR product spanning the left end of the largest duplication and the right end of the deleted region confirms the integration of the duplicated fragment within the deletion (Figure 4). Confirmation of the structure of the left end of the insertion, or elucidation of the position of the smaller duplicated region, could not be achieved leading to the conclusion that this region is physically complex.

### Accurate identification of lethal mutations positioned within the deficiency map

**Figure 5:**
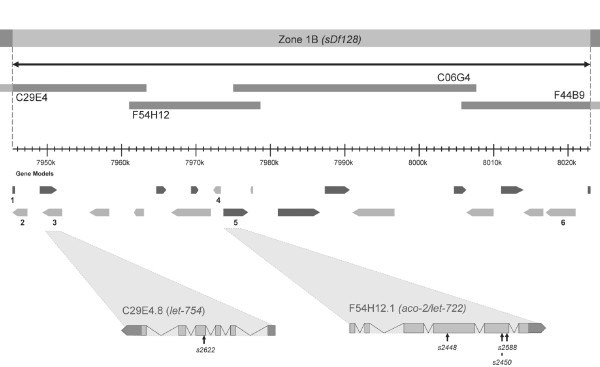
**Schematic representation of zone 1 B as defined by the deficiency *sDf128***. Cosmids as represented as grey bars above predicted gene positions taken from Wormbase (release WS170). Essential Gene candidates as defined by RNAi phenotype are numbered. Identified mutation positions are shown in gene expansions.

A more detailed analysis of Zone 1B highlights the value of applying the physical deficiency map to the identification of uncloned mutations. This zone is defined by the deletion breakpoints of the deficiency *sDf128 *and contains three molecularly unknown lethal mutants; *let-722*, *let-754 *and *let-766*. The region is 78 kb in size, contains 21 predicted genes and is spanned by the cosmids; C29E4, F54H12, C06G4 and F44B9 (Figure 5).

Since 39 of the 40 molecularly identified lethal mutants in Wormbase [18] exhibit a variety of lethal or sterile RNAi phenotypes (Additional file 1), genes within each zone which display these RNAi phenotypes make good candidates for the lethal mutations mapping to the same region. Of the 21 genes within zone 1 B, only 6 exhibit lethal phenotypes by RNAi (Figure 5 and Additional file 1) and these 6 genes were consequently taken as initial candidates of the three lethal mutants in this region.

To identify DNA lesions the coding region of each candidate gene was PCR amplified and sequenced using homozygous mutant DNA from a representative lethal strain as template. In this way both *let-722 *and *let-754*, were identified as being alleles of *aco-2 *and C29E4.8 respectively (summarized in Table 3). *let-766 *however was not identified from this approach and we conclude that this mutation either maps into one of the remaining 19 genes within this region, or a gene outside the deficiency which may have be disrupted through integration of the small unmapped duplication present in this strain.

**Table 3:** Summary of the mutations identified in essential genes as a result of this study.

Gene	Description	Genomic Position (bp)	Chromosome	Mutant	Allele	Mutation	Protein Change	RNAi Phenotype	Mutagen
C16A3.3	RRP5 Ribosomal RNA Processing Enzyme	6383494..6389343	III	*let-716*	*s2457*	G-A	3rd Intron Splice Site	emb	EMS
					*s2626*	TGG-TGA	W1489 *(opal)*		
*fum-1*	Fumarase	7465428..7467668	III	*let-768*	*s2482*	TCG-TTG	S149L	emb	EMS
					*s2592*	CGT-CAT	R151H		
					*s2628*	GGA-AGA	G88R		
C29E4.8	Adenylate Kinase	7951844..7949909	III	*let-754*	*s2622*	AAG-AAA	R150C	emb	EMS
*aco-2*	Aconitase	7973646..7977254	III	*let-722*	*s2448*	GAA-AAA	E285K	emb	EMS
					*s2450*	GGG-GGA	G663D		
					*s2588*	CGC-CGT	A687V		

emb = embryonic lethality

EMS = Ethylmethane Sulphonate

Finally, through the incorporation of mapping data generated by our group from ongoing efforts to clone lethal mutations, we have been able to further identify the mutations *let-716 *and *let-768 *as alleles of the genes C16A3.3 and *fum-1*, respectively (our unpublished data and summarized in Table 3).

## Conclusion

We have demonstrated that oaCGH can be successfully applied to the rapid and precise characterization of existing *C. elegans *genetic deficiencies. This study highlights how this type of analysis can transform low resolution genetic mapping tools into a precise physical mapping resource with which to accurately position molecularly unidentified mutations.

The implementation of oaCGH technology for this purpose is straightforward, requiring only the preparation of high quality genomic DNA from the deficiency strains of interest. Data is generated in a short time and can be verified experimentally by standard molecular techniques. Some caveats to the application of this methodology have been highlighted by the initial difficulties we describe resulting from the use of animals balanced by a free duplication, though we have demonstrated that by considered evaluation of the experimental approach these issues can be circumvented easily.

We envisage that this resource will be instrumental in resolving previously ambiguous genetic mapping data. Additionally, we propose that integration of a physical deficiency map into a high-throughput molecular mapping strategy such as snip-SNP will improve the efficiency of positional cloning in *C. elegans*. In such an approach snip-SNP would be employed to map the mutant of interest into a sub-chromosomal region, this region could then be further broken up by physically defined deficiencies, and a candidate sequencing pipeline, such as the one we describe here, employed to identify the molecular position of the mutation of interest.

## Methods

### Nematode culture, harvest, and DNA preparation

Nematodes were grown as previously described [19] on 150 mm NGM agar plates seeded with Escherichia coli strain OP50. Strains used: Bristol N2, JK2689 – *pop-1(q645) dpy-5(e61) I/hT2 [bli-4(e937) let-?(q782) qIs48] (I;III)*, BC4697 – *sDf121(s2098) unc-32(e189) III; sDp3 (III;f)*, BC7162 – *dpy-17(e164) sDf125(s2424) unc-32(e189) III; hT2 [bli-4(e937) let-?(q782) qIs48]*, BC4638 – *dpy-17(e164) sDf127(s2428) unc-32(e189) III; sDp3 (III;f)*, BC7163 – *dpy-17(e164) sDf127(s2428) unc-32(e189) III; hT2 [bli-4(e937) let-?(q782) qIs48]*, BC4690 – *dpy-17(e164) sDf128(s2786) unc-32(e189) III; sDp3 (III;f)*, BC7164 – *dpy-17(e164) sDf128(s2786) unc-32(e189) III; hT2 [bli-4(e937) let-?(q782) qIs48]*, BC7244 – *sDf130(s2427) unc-32(e189) III; hT2 [bli-4(e937) let-?(q782) qIs48]*, BC7181 – *dpy-17(e164) sDf135(s2767) unc-32(e189) III; hT2 [bli-4(e937) let-?(q782) qIs48]*, BC3737 – *sDp8(III;I); eT1(III;V)*, BC4158 *sDp3(III;f); dpy-17(e164) let-722(s2448) unc-32(e189) III*, BC4160 – *sDp3(III;f); dpy-17(e164) let-722(s2450) unc-32(e189) III*, BC4228 – *sDp3(III;f); dpy-17(e164) let-722(s2588) unc-32(e189)*, BC4262 – *sDp3(III;f); dpy-17(e164) let-754(s2622) unc-32(e189) III*, BC4173 – *dpy-17(e164) let-766(s2463) unc-32(e189) III; sDp3(III;f)*, BC4167 – *sDp3(III;f); dpy-17(e164) let-716(s2457) unc-32(e189) III*, BC4266 – *sDp3(III;f); dpy-17(e164) let-716(s2626) unc-32(e189) III*, BC4192 – *sDp3(III;f); dpy-17(e164) let-768(s2482) unc-32(e189) III*, BC4232 – *sDp3(III;f); dpy-17(e164) let-768(s2592) unc-32(e189) III*, BC4268 – *sDp3(III;f); dpy-17(e164) let-768(s2628) unc-32(e189) III*, BC2842 – *dpy-18(e364)/eT1 III; unc-46(e177) let-444(s1418)/eT1 [let-500(s2165)]V*, MT3022 – *nDf20/sma-2(e502) unc-32(e189)III*.

DNA preparation for oaCGH samples was done as previously described [13]. For mutant sequencing ~50 homozygous lethal animals were picked from the progeny of *sDp3 *balanced strains into 5 ul of lysis buffer (50 mM KCl, 10 mM Tris pH 8.3, 2.5 mM MgCl2, 0.45% Tween-20, 0.01% Gelatin) and freeze cracked in Liquid N2 followed by digestion (1 hour at 60°C followed by 15 min at 95°C). 1 ul of this DNA preparation was subsequently used in a 25 ul PCR reaction.

### oaCGH Data analysis

For the analysis of *sDp3 *balanced strains a *C. elegans *whole genome array was used, based on Wormbase release WS120, and available from NimbleGen Systems Inc. [20]. Analysis of the *hT2 (qIs48) *balanced strains was performed with a whole genome *C. elegans *array designed with overlapping 50-mer probes targeting primarily annotated exons and micro-RNAs [13]. oaCGH sample preparation, hybridization and analysis was done as previously described [13]. Copy number aberrations were detected by visual inspection using the SignalMap™ browser software [20]. The data discussed in this publication have been deposited in NCBIs Gene Expression Omnibus (GEO) [21] and are accessible through GEO Series accession numbers GSE9214 and GPL6047.

### Molecular identification of deficiency breakpoints and mutations

PCR amplification across the region of the breakpoint was performed with nested primers (Primer sequences and amplification conditions available upon request) and sequenced using standard molecular methods.

## Authors' contributions

MRJ carried out the experiments, data analysis and drafted the manuscript. JSM assisted in sample preparation. SF designed the oaCGH array. DGM helped to draft the manuscript. DLB conceived of the study, and participated in its design and coordination. All authors read and approved the final manuscript.

## Supplementary Material

Additional file 1 Identified mutants of essential genes and their associated RNAi phenotype. Click here for file
