# Supplementary material for: Oligonucleotide Array Comparative Genomic Hybridization (oaCGH) based characterization of genetic deficiencies as an aid to gene mapping in Caenorhabditis elegans
Source: BMC Genomics. 2007 Nov 7;8:402. doi: 10.1186/1471-2164-8-402 (PMC2220004; doi:10.1186/1471-2164-8-402)
Supplement: Additional file 1 — Identified mutants of essential genes and their associated RNAi phenotype. [file 1471-2164-8-402-S1.DOC]

**Table S1:** Identified mutants of essential genes and their associated RNAi phenotype

| **Gene Name** | **Start (bp)** | **Description** | **RNAi Phen.** |
| --- | --- | --- | --- |
| kle-2 (partial) | 7941545 | Kleisin Family | emb |
| C29E4.7 | 7945523 | Glutathione S-transferase | emb |
| col-90 | 7948766 | Collagen (type IV and XIII) | dpy |
| C29E4.8 | 7949252 | Adenylate kinase | emb |
| C29E4.11 | 7955777 | Pseudogene | none |
| C29E4.9 | 7956021 | Pseudogene | none |
| F54H12.3 | 7958601 | Transposon | none |
| F54H12.4 | 7961524 | None | none |
| F54H12.2 | 7964509 | None | none |
| F54H12.5 | 7966570 | F-Box containing protein | none |
| F54H12.7 | 7969289 | None | none |
| F54H12.6 | 7972334 | Elongation factor 1 beta/delta chain | emb |
| aco-2 | 7973645 | Aconitase | emb |
| C06G4.4 | 7977178 | None | none |
| C06G4.t1 | 7978399 | tRNA | none |
| clp-1 | 7980995 | calpain homolog | ced |
| C06G4.1 | 7987057 | Vigilin | none |
| C06G4.5 | 7990717 | G-protein coupled receptor | none |
| C06G4.6 | 8004388 | None | none |
| cit-1.1 | 8005863 | cyclin T ortholog | none |
| cit-1.2 | 8010706 | cyclin T ortholog | none |
| F44B9.5 | 8013740 | Predicted phosphate acyltransferase | none |
| lin-36 | 8016576 | Novel protein | lva |
| F44B9.2 (partial) | 8021958 | None | none |

**Table S2:** *sDf128* deleted genes and associated phenotype by RNAi.

| **Gene Name** | **RNAi Phenotype** | **Gene Name** | **RNAi Phenotype** |
| --- | --- | --- | --- |
|
| *let-49* | EMB | *let-8* | EMB |
| *let-502* | EMB | *let-17* | EMB |
| *let-363* | EMB | *let-21* | EMB |
| *let-381* | LVL | *let-29* | EMB |
| *let-75* | EMB | *let-76* | EMB |
| *let-268* | EMB | *let-77* | EMB |
| *let-858* | EMB | *let-94* | EMB |
| *let-19* | EMB | *let-328* | EMB |
| *let-23* | none | *let-333* | EMB |
| *let-805* | EMB | *let-341* | EMB |
| *let-767* | EMB | *let-354* | EMB |
| *let-756* | none | *let-412* | EMB |
| *let-721* | EMB | *let-425* | EMB |
| *let-711* | EMB | *let-461* | EMB |
| *let-70* | EMB | *let-512* | EMB |
| *let-60* | EMB | *let-553* | EMB |
| *let-92* | EMB | *let-603* | EMB |
| *let-653* | STE | *let-602* | STE |
| *let-99* | EMB | *let-740* | EMB |
| *let-418* | EMB | *let-7* | NA |
| *let-413* | EMB | *let-2* | EMB |

**Table S3:** Gene complement of Zones 1-14 and their associated lethal mutants.

| Zone | Lethal Mutants | Gene Candidates | Start (bp) | End (bp) |
| --- | --- | --- | --- | --- |
| **14** |  |  | **3115819** | |
| 14 | let-706 let-720 let-758 let-759 let-760 let-776 let-791 let-811 let-831 | Y53G8B.4 | 3112793 | 3127107 |
| 14 |  | Y53G8B.1 | 3129309 | 3131166 |
| 14 |  | mec-12 | 3131566 | 3139677 |
| 14 |  | mir-76 | 3142095 | 3142114 |
| 14 |  | C44B11.4 | 3142212 | 3143749 |
| 14 |  | C44B11.6 | 3152726 | 3153462 |
| 14 |  | C44B11.1 | 3155258 | 3158383 |
| 14 |  | R148.5 | 3166274 | 3171187 |
| 14 |  | R148.4 | 3171656 | 3172339 |
| 14 |  | R148.3 | 3175328 | 3183596 |
| 14 |  | R148.2 | 3183682 | 3184991 |
| 14 |  | xbx-7 | 3186689 | 3191360 |
| 14 |  | R148.t2 | 3186979 | 3187050 |
| 14 |  | R148.t3 | 3189658 | 3189731 |
| 14 |  | R148.t4 | 3191640 | 3191709 |
| 14 |  | R148.t1 | 3195428 | 3195509 |
| 14 |  | R148.7 | 3195577 | 3197357 |
| 14 |  | heh-1 | 3198016 | 3199507 |
| 14 |  | frm-8 | 3200789 | 3219141 |
| 14 |  | H09G03.1 | 3226341 | 3230641 |
| 14 |  | Y53G8AL.1 | 3233091 | 3241252 |
| 14 |  | Y53G8AL.2 | 3241712 | 3248568 |
| 14 |  | Y53G8AL.3 | 3251450 | 3253132 |
| 14 |  | Y53G8AM.4 | 3254015 | 3255608 |
| 14 |  | Y53G8AM.5 | 3256719 | 3258384 |
| 14 |  | Y53G8AM.6 | 3259320 | 3260871 |
| 14 |  | Y53G8AM.7 | 3263671 | 3266597 |
| 14 |  | Y53G8AM.8 | 3267167 | 3278687 |
| 14 |  | Y53G8AM.2 | 3271811 | 3272032 |
| 14 |  | Y53G8AR.7 | 3282297 | 3290190 |
| 14 |  | Y53G8AR.8 | 3290244 | 3294319 |
| 14 |  | Y53G8AR.6 | 3294741 | 3297155 |
| 14 |  | Y53G8AR.5 | 3297447 | 3301949 |
| 14 |  | ral-1 | 3311423 | 3317750 |
| 14 |  | Y53G8AR.9 | 3317734 | 3321234 |
| 14 |  | Y53G8AR.2 | 3322959 | 3331655 |
| 14 |  | Y53G8AR.1 | 3333025 | 3333946 |
| 14 |  | F45H7.t1 | 3339463 | 3339534 |
| 14 |  | F45H7.1 | 3344865 | 3348180 |
| 14 |  | gei-1 | 3355588 | 3363403 |
| 14 |  | prk-2 | 3363658 | 3370198 |
| 14 |  | F45H7.6 | 3377397 | 3384157 |
| 14 |  | C56G7.2 | 3386896 | 3388865 |
| 14 |  | C56G7.t1 | 3389524 | 3389596 |
| 14 |  | C56G7.3 | 3389908 | 3392504 |
| 14 |  | mlc-4 | 3392499 | 3393780 |
| 14 |  | F59A2.3 | 3396569 | 3397661 |
| 14 |  | F59A2.4 | 3397729 | 3400615 |
| 14 |  | npp-9 | 3400627 | 3404465 |
| 14 |  | F59A2.5 | 3404863 | 3405395 |
| 14 |  | F59A2.2 | 3405562 | 3411199 |
| 14 |  | F59A2.6 | 3415538 | 3424729 |
| 14 |  | K01A11.2 | 3425727 | 3427995 |
| 14 |  | K01A11.t1 | 3426900 | 3426972 |
| 14 |  | K01A11.1 | 3428407 | 3431338 |
| 14 |  | spe-41 | 3438998 | 3444196 |
| 14 |  | K01A11.3 | 3447125 | 3453969 |
| 14 |  | K01A11.5 | 3448551 | 3451084 |
| 14 |  | C34C12.1 | 3454330 | 3455835 |
| 14 |  | C34C12.2 | 3456462 | 3460065 |
| 14 |  | C34C12.8 | 3460391 | 3461575 |
| 14 |  | pph-6 | 3461688 | 3467647 |
| 14 |  | C34C12.4 | 3467651 | 3469733 |
| 14 |  | C34C12.5 | 3471988 | 3473692 |
| 14 |  | C34C12.9 | 3474240 | 3475434 |
| 14 |  | C34C12.6 | 3482575 | 3484827 |
| 14 |  | C34C12.7 | 3484845 | 3486026 |
| 14 |  | gly-14 | 3490403 | 3493216 |
| 14 |  | M01F1.9 | 3494262 | 3499041 |
| 14 |  | M01F1.10 | 3496971 | 3499041 |
| 14 |  | M01F1.8 | 3499374 | 3502522 |
| 14 |  | rpl-16 | 3502822 | 3503594 |
| 14 |  | M01F1.3 | 3502824 | 3505210 |
| 14 |  | M01F1.4 | 3505642 | 3510017 |
| 14 |  | M01F1.6 | 3510219 | 3510959 |
| 14 |  | M01F1.5 | 3510958 | 3519591 |
| 14 |  | M01F1.7 | 3521130 | 3529711 |
| 14 |  | rpl-37 | 3528403 | 3528952 |
| 14 |  | C54C6.6 | 3534775 | 3537453 |
| 14 |  | ben-1 | 3538277 | 3541592 |
| 14 |  | C54C6.7 | 3545747 | 3546399 |
| 14 |  | C54C6.5 | 3549323 | 3549822 |
| 14 |  | C54C6.4 | 3555069 | 3556468 |
| 14 |  | nhr-6 | 3563729 | 3578758 |
| 14 |  | C48D5.3 | 3578866 | 3581677 |
| 14 |  | ptp-1 | 3590718 | 3611975 |
| 14 |  | sel-8 | 3613200 | 3627122 |
| 14 |  | C32A3.2 | 3628339 | 3630540 |
| 14 |  | C32A3.3 | 3636025 | 3643638 |
| **14,13A** |  |  | **3640101** | |
| 13A | *let-700 let-710 let-715 let-736 let-737 let-738 let-763 let-796 let-812 let-813 let-819 let-820 mel-33* | C32A3.3 | 3636025 | 3643638 |
| 13A |  | unc-93 | 3644354 | 3649696 |
| 13A |  | C46F11.3 | 3648892 | 3650811 |
| 13A |  | C46F11.2 | 3650885 | 3653207 |
| 13A |  | C46F11.5 | 3652396 | 3657581 |
| 13A |  | C46F11.4 | 3657644 | 3664473 |
| 13A |  | C46F11.6 | 3667603 | 3670415 |
| 13A |  | T27D1.3 | 3683301 | 3685804 |
| 13A |  | cyn-9 | 3697868 | 3699612 |
| 13A |  | pdi-1 | 3699719 | 3701455 |
| 13A |  | C14B1.2 | 3702261 | 3703108 |
| 13A |  | C14B1.3 | 3703627 | 3705006 |
| 13A |  | tag-125 | 3705244 | 3706784 |
| 13A |  | C14B1.5 | 3706866 | 3709193 |
| 13A |  | C14B1.10 | 3709309 | 3713261 |
| 13A |  | C14B1.6 | 3713468 | 3718703 |
| 13A |  | C14B1.8 | 3718570 | 3719652 |
| 13A |  | C14B1.7 | 3720279 | 3723576 |
| 13A |  | C14B1.9 | 3724256 | 3726477 |
| 13A |  | F34D10.2 | 3726878 | 3735021 |
| 13A |  | F34D10.8 | 3730808 | 3731176 |
| 13A |  | F34D10.3 | 3734229 | 3734825 |
| 13A |  | F34D10.4 | 3736579 | 3739731 |
| 13A |  | lin-48 | 3745440 | 3747271 |
| 13A |  | F34D10.7 | 3747539 | 3752612 |
| 13A |  | F34D10.6 | 3753983 | 3758189 |
| 13A |  | C44F1.1 | 3760568 | 3763060 |
| 13A |  | C44F1.2 | 3768522 | 3770228 |
| 13A |  | lec-4 | 3771454 | 3773312 |
| 13A |  | acy-3 | 3774872 | 3781876 |
| 13A |  | mtm-3 | 3789963 | 3800444 |
| 13A |  | xbx-5 | 3802555 | 3804346 |
| 13A |  | toh-1 | 3804751 | 3809009 |
| 13A |  | dpy-27 | 3813532 | 3819656 |
| 13A |  | amx-1 | 3822382 | 3826240 |
| 13A |  | R13G10.4 | 3826357 | 3829184 |
| 13A |  | C36A4.10 | 3829809 | 3830214 |
| 13A |  | cyp-25A1 | 3832578 | 3834500 |
| 13A |  | cyp-25A2 | 3835199 | 3837128 |
| 13A |  | cyp-25A3 | 3837578 | 3839456 |
| 13A |  | C36A4.11 | 3840574 | 3841129 |
| 13A |  | C36A4.4 | 3841241 | 3843595 |
| 13A |  | C36A4.5 | 3844254 | 3848241 |
| 13A |  | cyp-25A4 | 3849886 | 3852050 |
| 13A |  | brc-1 | 3853123 | 3862937 |
| 13A |  | C36A4.9 | 3864015 | 3867076 |
| 13A |  | rpl-3 | 3867826 | 3869504 |
| 13A |  | tir-1 | 3869571 | 3901443 |
| 13A |  | mmcm-1 | 3903091 | 3907666 |
| 13A |  | pat-3 | 3908625 | 3914115 |
| 13A |  | tag-252 | 3916910 | 3918786 |
| 13A |  | ZK1058.3 | 3918848 | 3920676 |
| 13A |  | ZK1058.5 | 3920635 | 3921683 |
| 13A |  | nit-1 | 3922429 | 3923764 |
| 13A |  | ZK1058.9 | 3923769 | 3925455 |
| 13A |  | F37A8.1 | 3928434 | 3929105 |
| 13A |  | F37A8.2 | 3929528 | 3931402 |
| 13A |  | nlp-10 | 3933602 | 3935885 |
| 13A |  | F37A8.5 | 3956601 | 3957452 |
| 13A |  | R10E9.3 | 3960235 | 3961683 |
| 13A |  | R10E9.2 | 3962769 | 3964075 |
| 13A |  | msi-1 | 3964628 | 3976374 |
| 13A |  | Y1A5A.2 | 3984344 | 3984647 |
| 13A |  | Y1A5A.1 | 3985560 | 3986786 |
| 13A |  | C36E8.6 | 3988036 | 3988552 |
| 13A |  | C36E8.2 | 3989352 | 3992152 |
| 13A |  | C36E8.1 | 3999274 | 4004120 |
| 13A |  | C36E8.3 | 4006119 | 4009939 |
| 13A |  | C36E8.4 | 4012313 | 4015739 |
| 13A |  | tbb-2 | 4015738 | 4017619 |
| 13A |  | T02C12.2 | 4020038 | 4021751 |
| 13A |  | T02C12.3 | 4022139 | 4025595 |
| 13A |  | hum-5 | 4029088 | 4036813 |
| 13A |  | T02C12.4 | 4041196 | 4042465 |
| 13A |  | E03A3.1 | 4042643 | 4047604 |
| 13A |  | rcq-5 | 4052786 | 4057948 |
| 13A |  | his-69 | 4058332 | 4058715 |
| 13A |  | his-70 | 4059712 | 4060092 |
| 13A |  | E03A3.5 | 4060564 | 4062795 |
| 13A |  | unc-79 | 4069184 | 4074148 |
| 13A |  | E03A3.7 | 4074339 | 4075112 |
| 13A |  | E03A3.8 | 4077277 | 4077792 |
| 13A |  | C03C10.6 | 4078415 | 4082768 |
| 13A |  | kin-19 | 4083981 | 4086616 |
| 13A |  | C03C10.5 | 4090265 | 4090531 |
| 13A |  | C03C10.2 | 4090814 | 4093253 |
| 13A |  | rnr-2 | 4092861 | 4094898 |
| 13A |  | C03C10.7 | 4096018 | 4096875 |
| 13A |  | C03C10.4 | 4096874 | 4098101 |
| 13A |  | C30D11.3 | 4121437 | 4122609 |
| 13A |  | C30D11.2 | 4128474 | 4129766 |
| 13A |  | unc-103 | 4138685 | 4147228 |
| 13A |  | C16C10.12 | 4147427 | 4151512 |
| 13A |  | C16C10.11 | 4152025 | 4152874 |
| 13A |  | tag-73 | 4153574 | 4154559 |
| 13A |  | C16C10.9 | 4155361 | 4157353 |
| 13A |  | C16C10.8 | 4161541 | 4164145 |
| 13A |  | rnf-5 | 4164306 | 4166808 |
| 13A |  | C16C10.6 | 4166917 | 4168738 |
| 13A |  | C16C10.5 | 4168809 | 4171134 |
| 13A |  | C16C10.4 | 4171309 | 4172151 |
| 13A |  | C16C10.3 | 4172307 | 4176132 |
| 13A |  | C16C10.13 | 4176657 | 4177466 |
| 13A |  | C16C10.2 | 4177604 | 4178639 |
| 13A |  | C16C10.1 | 4179035 | 4181413 |
| 13A |  | lrs-1 | 4181062 | 4186796 |
| 13A |  | R74.2 | 4187130 | 4189974 |
| 13A |  | dnj-16 | 4191114 | 4193131 |
| 13A |  | xbp-1 | 4193794 | 4195321 |
| 13A |  | asd-1 | 4197256 | 4203412 |
| 13A |  | R74.6 | 4202347 | 4204242 |
| 13A |  | R74.7 | 4204402 | 4205787 |
| 13A |  | R74.8 | 4206250 | 4208195 |
| 13A |  | F43C1.1 | 4210535 | 4216678 |
| 13A |  | mpk-1 | 4216736 | 4228066 |
| 13A |  | F43C1.5 | 4229169 | 4230365 |
| 13A |  | nhr-20 | 4230406 | 4233191 |
| 13A |  | F43C1.7 | 4241797 | 4242527 |
| 13A |  | F43C1.6 | 4242664 | 4243535 |
| 13A |  | F43C1.3 | 4243656 | 4244357 |
| 13A |  | Y44F5A.1 | 4244829 | 4247643 |
| 13A |  | T08A11.1 | 4248134 | 4263020 |
| 13A |  | T08A11.2 | 4263771 | 4269242 |
| 13A |  | R10E4.1 | 4269734 | 4272417 |
| 13A |  | tag-310 | 4276175 | 4280191 |
| 13A |  | R10E4.3 | 4285833 | 4287444 |
| 13A |  | mcm-5 | 4288236 | 4291255 |
| 13A |  | nth-1 | 4291656 | 4293268 |
| 13A |  | R10E4.6 | 4293380 | 4294897 |
| 13A |  | R10E4.7 | 4295255 | 4296479 |
| 13A |  | R10E4.11 | 4299413 | 4300996 |
| 13A |  | R10E4.9 | 4301650 | 4302353 |
| 13A |  | evl-14 | 4303076 | 4313096 |
| 13A |  | dcn-1 | 4313595 | 4317549 |
| 13A |  | H38K22.8 | 4317311 | 4317353 |
| 13A |  | tag-131 | 4317674 | 4320300 |
| 13A |  | H38K22.6 | 4320427 | 4321293 |
| 13A |  | H38K22.7 | 4321720 | 4322839 |
| 13A |  | H38K22.4 | 4322941 | 4323438 |
| 13A |  | gly-6 | 4324258 | 4331199 |
| 13A |  | B0285.1 | 4333368 | 4340435 |
| 13A |  | B0285.3 | 4342017 | 4343307 |
| 13A |  | B0285.4 | 4343432 | 4345193 |
| 13A |  | hse-5 | 4345310 | 4350029 |
| 13A |  | B0285.11 | 4350796 | 4352136 |
| 13A |  | B0285.6 | 4352217 | 4355944 |
| 13A |  | B0285.7 | 4356510 | 4364943 |
| 13A |  | B0285.t1 | 4362495 | 4362614 |
| 13A |  | ckb-1 | 4365411 | 4367362 |
| 13A |  | ckb-2 | 4367803 | 4370070 |
| 13A |  | B0285.12 | 4369446 | 4369738 |
| 13A |  | ckb-3 | 4370856 | 4372791 |
| 13A |  | B0284.5 | 4373500 | 4374948 |
| 13A |  | B0284.1 | 4376474 | 4378804 |
| 13A |  | B0284.6 | 4378878 | 4379170 |
| 13A |  | B0284.3 | 4379339 | 4381012 |
| 13A |  | B0284.2 | 4381710 | 4384117 |
| 13A |  | B0284.4 | 4384758 | 4387728 |
| 13A |  | R07E5.4 | 4394767 | 4396297 |
| 13A |  | R07E5.5 | 4397595 | 4398456 |
| 13A |  | R07E5.6 | 4398784 | 4400869 |
| 13A |  | R07E5.15 | 4401429 | 4402421 |
| 13A |  | R07E5.17 | 4402577 | 4404294 |
| 13A |  | R07E5.3 | 4404757 | 4407233 |
| 13A |  | rnp-4 | 4406251 | 4407297 |
| 13A |  | prdx-3 | 4407413 | 4408408 |
| 13A |  | R07E5.1 | 4408522 | 4412120 |
| 13A |  | R07E5.13 | 4413428 | 4414725 |
| 13A |  | R07E5.16 | 4414156 | 4414257 |
| 13A |  | R07E5.11 | 4414811 | 4415197 |
| 13A |  | pdcd-2 | 4415509 | 4418028 |
| 13A |  | R07E5.7 | 4418314 | 4419140 |
| 13A |  | cku-80 | 4419819 | 4423296 |
| 13A |  | C28A5.6 | 4423750 | 4429470 |
| 13A |  | C28A5.7 | 4426825 | 4426926 |
| 13A |  | C28A5.t3 | 4428924 | 4428995 |
| 13A |  | C28A5.1 | 4429589 | 4434315 |
| 13A |  | C28A5.2 | 4434732 | 4439514 |
| 13A |  | nex-3 | 4442052 | 4444090 |
| 13A |  | ceh-43 | 4446609 | 4449911 |
| 13A |  | C28A5.t2 | 4449501 | 4449573 |
| 13A |  | C28A5.t1 | 4450292 | 4450364 |
| 13A |  | C28A5.5 | 4450905 | 4452116 |
| 13A |  | F56F3.3 | 4458447 | 4459430 |
| 13A |  | ndg-4 | 4462824 | 4467345 |
| 13A |  | F56F3.4 | 4468846 | 4470713 |
| 13A |  | F56F3.t1 | 4470854 | 4470926 |
| 13A |  | pqn-45 | 4471378 | 4475111 |
| 13A |  | rps-1 | 4475886 | 4477093 |
| 13A |  | ins-17 | 4476856 | 4477551 |
| 13A |  | cej-1 | 4489325 | 4491365 |
| 13A |  | atf-7 | 4491600 | 4499449 |
| 13A |  | cct-5 | 4499159 | 4508083 |
| 13A |  | F25F2.1 | 4508829 | 4512094 |
| 13A |  | cdh-4 | 4520426 | 4536956 |
| 13A |  | ugt-62 | 4537244 | 4540604 |
| 13A |  | M88.2 | 4541412 | 4542419 |
| 13A |  | M88.7 | 4542848 | 4546539 |
| 13A |  | M88.3 | 4543590 | 4544941 |
| 13A |  | M88.4 | 4549030 | 4551472 |
| 13A |  | M88.5 | 4553680 | 4560381 |
| 13A |  | pan-1 | 4562705 | 4566239 |
| 13A |  | F35G12.1 | 4566250 | 4568097 |
| 13A |  | F35G12.2 | 4567292 | 4572756 |
| **13A,12** |  |  | **4569043** | |
| 12 | *let-707* *let-732 let-733 let-734* *let-741* *let-761 let-778* *let-782 let-822* *let-823 let-824* *let-830* *let-836 let-840* | sel-5 | 4571728 | 4578404 |
| 12 |  | F35G12.4 | 4578820 | 4581649 |
| 12 |  | F35G12.12 | 4581760 | 4583852 |
| 12 |  | F35G12.5 | 4584899 | 4586441 |
| 12 |  | F35G12.7 | 4586644 | 4587266 |
| 12 |  | smc-4 | 4587473 | 4593076 |
| 12 |  | F35G12.11 | 4593143 | 4593794 |
| 12 |  | apc-11 | 4593739 | 4594389 |
| 12 |  | asb-1 | 4594510 | 4595911 |
| 12 |  | F10F2.1 | 4596109 | 4612302 |
| 12 |  | mab-21 | 4600704 | 4603019 |
| 12 |  | F10F2.3 | 4616840 | 4618739 |
| 12 |  | F10F2.2 | 4618850 | 4643247 |
| 12 |  | F10F2.4 | 4623019 | 4626125 |
| 12 |  | F10F2.7 | 4628412 | 4631053 |
| 12 |  | F10F2.6 | 4632373 | 4634394 |
| 12 |  | F10F2.8 | 4635296 | 4637456 |
| 12 |  | F10F2.5 | 4638396 | 4640527 |
| 12 |  | pqn-29 | 4643964 | 4646311 |
| 12 |  | T23F11.6 | 4646530 | 4647801 |
| 12 |  | T23F11.1 | 4650359 | 4653887 |
| 12 |  | T23F11.2 | 4654310 | 4655433 |
| 12 |  | cdka-1 | 4658704 | 4662640 |
| 12 |  | T23F11.4 | 4663216 | 4665324 |
| 12 |  | srg-13 | 4672214 | 4674129 |
| 12 |  | srg-10 | 4677731 | 4679628 |
| 12 |  | srg-11 | 4679978 | 4681527 |
| 12 |  | T04A8.3 | 4682739 | 4685499 |
| 12 |  | tag-243 | 4686077 | 4687160 |
| 12 |  | T04A8.5 | 4689270 | 4692087 |
| 12 |  | T04A8.17 | 4689598 | 4689735 |
| 12 |  | T04A8.6 | 4691077 | 4693263 |
| 12 |  | T04A8.8 | 4693959 | 4695167 |
| 12 |  | T04A8.7 | 4695325 | 4697972 |
| 12 |  | dnj-18 | 4697160 | 4699351 |
| 12 |  | sel-13 | 4699384 | 4700188 |
| 12 |  | clp-2 | 4700299 | 4704018 |
| 12 |  | T04A8.11 | 4704368 | 4705368 |
| 12 |  | tag-189 | 4705349 | 4706756 |
| 12 |  | T04A8.13 | 4707118 | 4712491 |
| 12 |  | T04A8.15 | 4712536 | 4715152 |
| 12 |  | emb-5 | 4715257 | 4720629 |
| 12 |  | acy-1 | 4722176 | 4730834 |
| 12 |  | F17C8.3 | 4731902 | 4735904 |
| 12 |  | F17C8.7 | 4736034 | 4737170 |
| 12 |  | ras-2 | 4737566 | 4740041 |
| 12 |  | F17C8.8 | 4741782 | 4742701 |
| 12 |  | twk-6 | 4742932 | 4744613 |
| 12 |  | F17C8.6 | 4744834 | 4745989 |
| 12 |  | col-89 | 4748144 | 4749120 |
| 12 |  | VB0393L.2 | 4749758 | 4750250 |
| 12 |  | B0393.8 | 4751842 | 4752334 |
| 12 |  | rps-0 | 4752963 | 4754047 |
| 12 |  | rbg-3 | 4754396 | 4756977 |
| 12 |  | B0393.3 | 4757212 | 4759668 |
| 12 |  | B0393.4 | 4759728 | 4763573 |
| 12 |  | B0393.5 | 4769041 | 4776241 |
| 12 |  | B0393.7 | 4776796 | 4778020 |
| 12 |  | B0393.9 | 4778355 | 4779986 |
| 12 |  | B0393.6 | 4780403 | 4781945 |
| 12 |  | C38D4.10 | 4782475 | 4782699 |
| 12 |  | C38D4.1 | 4783225 | 4785226 |
| 12 |  | mel-28 | 4786044 | 4792344 |
| 12 |  | C38D4.4 | 4793050 | 4795362 |
| 12 |  | tag-325 | 4796803 | 4802861 |
| 12 |  | C38D4.9 | 4802968 | 4804711 |
| 12 |  | pal-1 | 4804214 | 4808675 |
| 12 |  | C38D4.7 | 4810085 | 4811125 |
| 12 |  | arl-6 | 4813272 | 4814777 |
| 12 |  | F26A1.6 | 4816003 | 4817041 |
| 12 |  | F26A1.4 | 4818131 | 4818985 |
| 12 |  | F26A1.7 | 4819291 | 4819906 |
| 12 |  | F26A1.8 | 4820761 | 4822189 |
| 12 |  | F26A1.9 | 4825422 | 4826203 |
| 12 |  | F26A1.3 | 4826872 | 4828884 |
| 12 |  | nspd-9 | 4830019 | 4830293 |
| 12 |  | acbp-7 | 4837698 | 4840987 |
| 12 |  | F26A1.11 | 4841002 | 4843019 |
| 12 |  | F26A1.12 | 4843407 | 4844373 |
| 12 |  | fkh-5 | 4844625 | 4846509 |
| 12 |  | F26A1.13 | 4847035 | 4848930 |
| 12 |  | F26A1.14 | 4849229 | 4849813 |
| 12 |  | F26A1.1 | 4850471 | 4851860 |
| 12 |  | ced-4 | 4852288 | 4855129 |
| 12 |  | C35D10.8 | 4855290 | 4856035 |
| 12 |  | C35D10.7 | 4856156 | 4859192 |
| 12 |  | C35D10.6 | 4859302 | 4860649 |
| 12 |  | C35D10.10 | 4860644 | 4861757 |
| 12 |  | C35D10.5 | 4862521 | 4863238 |
| 12 |  | coq-8 | 4863390 | 4866912 |
| 12 |  | C35D10.17 | 4867025 | 4867432 |
| 12 |  | C35D10.11 | 4868297 | 4868732 |
| 12 |  | C35D10.3 | 4869010 | 4870059 |
| 12 |  | C35D10.2 | 4870741 | 4872012 |
| 12 |  | C35D10.12 | 4872530 | 4874714 |
| 12 |  | C35D10.13 | 4877680 | 4878273 |
| 12 |  | arx-6 | 4878626 | 4879365 |
| 12 |  | C35D10.1 | 4879708 | 4881414 |
| 12 |  | clec-5 | 4881915 | 4883541 |
| 12 |  | clec-6 | 4885660 | 4887405 |
| 12 |  | F26F4.5 | 4887884 | 4891215 |
| 12 |  | F26F4.6 | 4891582 | 4893372 |
| 12 |  | nhl-2 | 4894610 | 4899048 |
| 12 |  | tag-340 | 4899677 | 4903106 |
| 12 |  | kbp-2 | 4903247 | 4903669 |
| 12 |  | F26F4.8 | 4903977 | 4905329 |
| 12 |  | rom-1 | 4907189 | 4908785 |
| 12 |  | F26F4.12 | 4908945 | 4910001 |
| 12 |  | F26F4.9 | 4910446 | 4912450 |
| 12 |  | F26F4.2 | 4911224 | 4911955 |
| 12 |  | F26F4.1 | 4912170 | 4914033 |
| 12 |  | rrt-1 | 4913866 | 4917092 |
| 12 |  | F26F4.11 | 4917194 | 4917973 |
| 12 |  | ula-1 | 4918240 | 4920284 |
| 12 |  | mmab-1 | 4920396 | 4921617 |
| 12 |  | set-2 | 4922409 | 4930167 |
| 12 |  | C26E6.12 | 4930627 | 4932152 |
| 12 |  | C26E6.7 | 4932440 | 4934881 |
| 12 |  | C26E6.6 | 4935540 | 4937168 |
| 12 |  | fsn-1 | 4937301 | 4939393 |
| 12 |  | rpb-2 | 4939529 | 4944366 |
| 12 |  | C26E6.3 | 4944473 | 4947268 |
| 12 |  | C26E6.2 | 4952085 | 4955075 |
| 12 |  | C26E6.1 | 4956852 | 4957500 |
| 12 |  | C27F2.4 | 4958499 | 4960263 |
| 12 |  | C27F2.5 | 4960386 | 4963002 |
| 12 |  | C27F2.9 | 4961841 | 4963054 |
| 12 |  | nca-2 | 4965533 | 4979692 |
| 12 |  | C27F2.6 | 4979759 | 4980381 |
| 12 |  | C27F2.7 | 4980607 | 4984438 |
| 12 |  | C27F2.10 | 4985049 | 4986895 |
| 12 |  | C27F2.1 | 4987232 | 4991359 |
| 12 |  | C27F2.8 | 4991411 | 4998125 |
| 12 |  | R144.5 | 4999347 | 5001294 |
| 12 |  | R144.10 | 5001333 | 5002274 |
| 12 |  | R144.6 | 5003228 | 5004685 |
| 12 |  | R144.12 | 5004800 | 5006945 |
| 12 |  | R144.11 | 5007070 | 5008794 |
| 12 |  | larp-1 | 5009678 | 5017613 |
| 12 |  | wip-1 | 5018611 | 5021093 |
| 12 |  | mdt-11 | 5021189 | 5021859 |
| 12 |  | R144.3 | 5022008 | 5023070 |
| 12 |  | R144.13 | 5023287 | 5023937 |
| 12 |  | R144.2 | 5025332 | 5029598 |
| 12 |  | klp-6 | 5030893 | 5037589 |
| 12 |  | C45G9.6 | 5036634 | 5040365 |
| 12 |  | C45G9.13 | 5042389 | 5044635 |
| 12 |  | C45G9.14 | 5045060 | 5045616 |
| 12 |  | C45G9.7 | 5046133 | 5047105 |
| 12 |  | C45G9.5 | 5048080 | 5050628 |
| 12 |  | C45G9.8 | 5050133 | 5054232 |
| 12 |  | C45G9.9 | 5054605 | 5055717 |
| 12 |  | C45G9.10 | 5056822 | 5061850 |
| 12 |  | C45G9.4 | 5062668 | 5063781 |
| 12 |  | C45G9.11 | 5063955 | 5068686 |
| 12 |  | C45G9.2 | 5069030 | 5070270 |
| 12 |  | C45G9.12 | 5070392 | 5071558 |
| 12 |  | C45G9.1 | 5074659 | 5075929 |
| 12 |  | alh-1 | 5077724 | 5080626 |
| 12 |  | tag-174 | 5081685 | 5083352 |
| 12 |  | cah-1 | 5083167 | 5085250 |
| 12 |  | F54D8.p1 | 5098421 | 5098462 |
| 12 |  | F54D8.6 | 5101946 | 5106914 |
| **12,11** |  | **dpy-17** | **5107287** | **5108460** |
| 11 | *let-719 let-727 let-829* *let-753* *let-784 let-825 let-832* | sax-2 | 5109660 | 5130622 |
| 11 |  | tbx-2 | 5130886 | 5136278 |
| 11 |  | Y54H5A.2 | 5137281 | 5149541 |
| 11 |  | tag-262 | 5152000 | 5153113 |
| 11 |  | Y54H5A.1 | 5153423 | 5155383 |
| 11 |  | Y54H5A.4 | 5155463 | 5158734 |
| 11 |  | T10F2.5 | 5158870 | 5159563 |
| 11 |  | grs-1 | 5159483 | 5163586 |
| 11 |  | T10F2.2 | 5163898 | 5165250 |
| 11 |  | ulp-1 | 5165627 | 5169758 |
| 11 |  | T10F2.4 | 5169894 | 5172006 |
| 11 |  | K10D2.5 | 5172694 | 5173295 |
| 11 |  | K10D2.4 | 5173398 | 5173770 |
| 11 |  | cid-1 | 5173904 | 5180464 |
| 11 |  | K10D2.2 | 5185877 | 5188584 |
| 11 |  | K10D2.1 | 5188696 | 5196644 |
| 11 |  | emb-8 | 5196013 | 5199638 |
| 11 |  | K10D2.7 | 5196021 | 5196622 |
| 11 |  | ZC155.3 | 5203945 | 5206870 |
| 11 |  | syn-16 | 5207582 | 5209162 |
| 11 |  | ZC155.4 | 5211966 | 5214964 |
| 11 |  | ZC155.5 | 5215959 | 5217563 |
| 11 |  | ZC155.2 | 5219264 | 5220007 |
| 11 |  | nex-1 | 5225580 | 5226902 |
| 11 |  | C34E10.10 | 5226777 | 5227648 |
| 11 |  | atp-2 | 5228370 | 5230503 |
| 11 |  | tag-251 | 5230514 | 5234455 |
| 11 |  | C34E10.11 | 5234577 | 5236323 |
| 11 |  | wrs-2 | 5236369 | 5239987 |
| 11 |  | cnd-1 | 5240694 | 5242067 |
| 11 |  | C34E10.8 | 5249047 | 5254150 |
| 11 |  | gop-1 | 5254429 | 5258881 |
| 11 |  | gop-2 | 5258980 | 5260469 |
| 11 |  | gop-3 | 5260651 | 5263796 |
| 11 |  | C34E10.9 | 5262673 | 5263292 |
| 11 |  | hap-1 | 5263902 | 5264955 |
| 11 |  | gro-1 | 5265072 | 5267169 |
| 11 |  | ZC395.5 | 5267768 | 5268540 |
| 11 |  | ztf-8 | 5268641 | 5273430 |
| 11 |  | ZC395.4 | 5270220 | 5270970 |
| 11 |  | ZC395.10 | 5273760 | 5274728 |
| 11 |  | toc-1 | 5275184 | 5277756 |
| 11 |  | clk-1 | 5277853 | 5279292 |
| 11 |  | ZC395.11 | 5286045 | 5286264 |
| 11 |  | T15B12.1 | 5288139 | 5298311 |
| 11 |  | T15B12.t1 | 5294938 | 5295009 |
| 11 |  | T15B12.2 | 5300001 | 5301834 |
| 11 |  | F52C9.5 | 5302177 | 5306283 |
| 11 |  | F52C9.t1 | 5305864 | 5305935 |
| 11 |  | F52C9.7 | 5308559 | 5311099 |
| 11 |  | pqe-1 | 5311205 | 5321691 |
| 11 |  | F52C9.3 | 5321848 | 5326230 |
| 11 |  | F52C9.1 | 5326582 | 5333571 |
| 11 |  | ZK121.2 | 5341648 | 5344204 |
| 11 |  | glrx-21 | 5345240 | 5346075 |
| 11 |  | Y32H12A.2 | 5346096 | 5349354 |
| 11 |  | Y32H12A.9 | 5352799 | 5353147 |
| 11 |  | Y32H12A.1 | 5362167 | 5364315 |
| 11 |  | dhs-9 | 5364479 | 5368004 |
| 11 |  | Y32H12A.4 | 5369141 | 5371234 |
| 11 |  | Y32H12A.5 | 5375660 | 5379888 |
| 11 |  | Y32H12A.6 | 5380929 | 5381959 |
| 11 |  | Y32H12A.7 | 5382228 | 5385373 |
| 11 |  | Y32H12A.8 | 5385484 | 5404943 |
| 11 |  | W03A5.2 | 5406094 | 5407926 |
| 11 |  | W03A5.1 | 5408068 | 5413135 |
| 11 |  | grl-22 | 5413181 | 5413951 |
| 11 |  | dnj-24 | 5416093 | 5419523 |
| 11 |  | W03A5.4 | 5431365 | 5434425 |
| 11 |  | W03A5.5 | 5438784 | 5439910 |
| 11 |  | W03A5.6 | 5440071 | 5440590 |
| 11 |  | T24G10.2 | 5440732 | 5442713 |
| 11 |  | F48E8.6 | 5443264 | 5446453 |
| 11 |  | skpt-1 | 5446479 | 5448376 |
| 11 |  | F48E8.8 | 5448795 | 5449584 |
| 11 |  | paa-1 | 5450063 | 5453569 |
| 11 |  | F48E8.4 | 5453563 | 5458926 |
| 11 |  | F48E8.3 | 5461264 | 5464328 |
| 11 |  | F48E8.2 | 5464355 | 5467930 |
| 11 |  | lon-1 | 5474436 | 5477971 |
| 11 |  | R02F2.6 | 5478640 | 5478882 |
| 11 |  | R02F2.5 | 5479362 | 5480236 |
| 11 |  | R02F2.7 | 5480271 | 5483742 |
| 11 |  | R02F2.4 | 5484158 | 5485898 |
| 11 |  | R02F2.2 | 5487600 | 5498901 |
| 11 |  | R02F2.8 | 5492908 | 5495226 |
| 11 |  | R02F2.9 | 5499182 | 5499899 |
| 11 |  | R02F2.1 | 5501390 | 5506354 |
| 11 |  | D1044.7 | 5507008 | 5508696 |
| 11 |  | pqn-25 | 5509167 | 5512560 |
| 11 |  | D1044.8 | 5512884 | 5517480 |
| 11 |  | C05D2.6 | 5516598 | 5603610 |
| 11 |  | D1044.4 | 5521676 | 5521987 |
| 11 |  | D1044.5 | 5524138 | 5524396 |
| 11 |  | D1044.2 | 5526028 | 5535437 |
| 11 |  | D1044.6 | 5535631 | 5540738 |
| 11 |  | D1044.1 | 5544260 | 5546243 |
| 11 |  | F09F7.5 | 5547024 | 5551198 |
| 11 |  | F09F7.6 | 5551278 | 5552071 |
| 11 |  | F09F7.7 | 5553468 | 5554890 |
| 11 |  | F09F7.4 | 5555360 | 5557187 |
| 11 |  | F09F7.3 | 5557300 | 5564787 |
| 11 |  | mlc-3 | 5564981 | 5567338 |
| **11,10** |  |  | **5565581** | |
| 10 |  | mlc-3 | 5564981 | 5567338 |
| 10 |  | nspb-12 | 5568166 | 5568734 |
| 10 |  | F09F7.1 | 5573950 | 5576640 |
| 10 |  | F56D2.5 | 5578390 | 5581175 |
| 10 |  | F56D2.8 | 5581537 | 5582366 |
| 10 |  | uev-2 | 5583096 | 5584168 |
| 10 |  | F56D2.3 | 5584642 | 5586933 |
| 10 |  | F56D2.2 | 5587218 | 5589836 |
| 10 |  | F56D2.6 | 5588991 | 5591674 |
| 10 |  | ucr-1 | 5592088 | 5594349 |
| 10 |  | ced-6 | 5595724 | 5598542 |
| 10 |  | C05D2.10 | 5597751 | 5600014 |
| 10 |  | gak-1 | 5604414 | 5607758 |
| 10 |  | C05D2.8 | 5606896 | 5610300 |
| 10 |  | bas-1 | 5612228 | 5614608 |
| 10 |  | C05D2.3 | 5614926 | 5617203 |
| 10 |  | daf-4 | 5624970 | 5632374 |
| 10 |  | C05D2.11 | 5626898 | 5628160 |
| 10 |  | ckk-1 | 5635433 | 5654510 |
| **10,13B** |  |  | **5637312** | |
| 13B |  | ckk-1 | 5635433 | 5654510 |
| 13B |  | C05H8.3 | 5653818 | 5653920 |
| 13B |  | sdz-21 | 5655174 | 5656946 |
| 13B |  | F54E7.6 | 5659841 | 5661037 |
| 13B |  | par-3 | 5664186 | 5676683 |
| **13B,9** |  |  | **5662800** | **5664335** |
| 9 | *let-718 let-780 let-797 let-798 let-799 let-809 let-810* | par-3 | 5664186 | 5676683 |
| 9 |  | F54E7.9 | 5676314 | 5677843 |
| 9 |  | F54E7.8 | 5677904 | 5678985 |
| 9 |  | rps-12 | 5679244 | 5680136 |
| 9 |  | F54E7.10 | 5679329 | 5679460 |
| 9 |  | F54E7.11 | 5679696 | 5679826 |
| 9 |  | pst-2 | 5680449 | 5682164 |
| 9 |  | rcn-1 | 5682297 | 5684049 |
| 9 |  | B0336.6 | 5690058 | 5692691 |
| 9 |  | B0336.5 | 5692867 | 5693844 |
| 9 |  | B0336.13 | 5693987 | 5694630 |
| 9 |  | B0336.7 | 5694686 | 5697316 |
| 9 |  | lgg-3 | 5697500 | 5697960 |
| 9 |  | rgs-5 | 5698386 | 5702321 |
| 9 |  | swp-1 | 5701227 | 5705415 |
| 9 |  | rpl-23 | 5705472 | 5706080 |
| 9 |  | B0336.11 | 5706688 | 5709280 |
| 9 |  | B0336.12 | 5710013 | 5710460 |
| 9 |  | B0336.3 | 5711499 | 5714738 |
| 9 |  | arf-1.2 | 5716064 | 5717534 |
| 9 |  | wrm-1 | 5722795 | 5727052 |
| 9 |  | egg-1 | 5728130 | 5730621 |
| 9 |  | B0244.9 | 5729343 | 5730961 |
| 9 |  | B0244.7 | 5731451 | 5732804 |
| 9 |  | B0244.10 | 5732982 | 5737054 |
| 9 |  | B0244.6 | 5737958 | 5744770 |
| 9 |  | B0244.5 | 5744829 | 5746999 |
| 9 |  | B0244.4 | 5747435 | 5751558 |
| 9 |  | ida-1 | 5755489 | 5765766 |
| 9 |  | B0244.t3 | 5762006 | 5762075 |
| 9 |  | B0244.t1 | 5762235 | 5762305 |
| 9 |  | B0244.11 | 5767833 | 5768616 |
| 9 |  | B0244.t2 | 5769855 | 5769936 |
| 9 |  | Y37B11A.1 | 5770136 | 5774534 |
| 9 |  | Y37B11A.2 | 5774633 | 5781970 |
| 9 |  | Y37B11A.t2 | 5782187 | 5782257 |
| 9 |  | Y37B11A.3 | 5782687 | 5785085 |
| 9 |  | polq-1 | 5791122 | 5802993 |
| 9 |  | ceh-10 | 5799024 | 5801000 |
| 9 |  | him-10 | 5803211 | 5805032 |
| 9 |  | R12B2.3 | 5805050 | 5806888 |
| 9 |  | R12B2.2 | 5807770 | 5809078 |
| 9 |  | R12B2.7 | 5814997 | 5815465 |
| 9 |  | sma-4 | 5816155 | 5820152 |
| 9 |  | R12B2.8 | 5823909 | 5828388 |
| 9 |  | mdt-15 | 5828511 | 5833697 |
| 9 |  | R12B2.6 | 5833974 | 5835708 |
| 9 |  | W04D12.1 | 5836878 | 5837135 |
| 9 |  | F01F1.7 | 5848808 | 5853216 |
| 9 |  | cct-6 | 5853484 | 5855645 |
| 9 |  | alh-9 | 5855990 | 5859130 |
| 9 |  | dpf-4 | 5859260 | 5862419 |
| 9 |  | F01F1.9 | 5860462 | 5863498 |
| 9 |  | rabn-5 | 5864498 | 5866408 |
| 9 |  | F01F1.10 | 5865570 | 5868451 |
| 9 |  | F01F1.15 | 5868547 | 5869572 |
| 9 |  | F01F1.11 | 5869636 | 5872348 |
| 9 |  | F01F1.3 | 5873006 | 5875316 |
| 9 |  | F01F1.12 | 5874216 | 5876496 |
| 9 |  | F01F1.13 | 5877119 | 5878370 |
| 9 |  | F01F1.2 | 5878472 | 5879285 |
| 9 |  | F01F1.1 | 5879440 | 5882383 |
| 9 |  | F01F1.14 | 5882333 | 5882874 |
| 9 |  | C28H8.5 | 5884258 | 5885726 |
| 9 |  | C28H8.7 | 5885798 | 5886907 |
| 9 |  | C28H8.8 | 5887055 | 5888726 |
| 9 |  | tag-327 | 5889708 | 5894179 |
| 9 |  | C28H8.13 | 5895193 | 5895957 |
| 9 |  | C28H8.9 | 5903523 | 5906073 |
| 9 |  | C28H8.4 | 5907000 | 5908374 |
| 9 |  | C28H8.3 | 5908649 | 5915104 |
| 9 |  | C28H8.11 | 5917591 | 5920037 |
| 9 |  | C28H8.2 | 5923138 | 5925007 |
| 9 |  | dnc-2 | 5924345 | 5925705 |
| 9 |  | C28H8.1 | 5926037 | 5926935 |
| 9 |  | zmp-1 | 5926849 | 5932904 |
| 9 |  | mir-67 | 5931319 | 5931338 |
| 9 |  | EGAP1.1 | 5935700 | 5937069 |
| 9 |  | ubq-1 | 5940193 | 5943784 |
| 9 |  | F25B5.5 | 5942840 | 5946074 |
| 9 |  | F25B5.6 | 5946176 | 5948660 |
| 9 |  | F25B5.3 | 5948839 | 5953472 |
| 9 |  | F25B5.7 | 5953658 | 5958193 |
| 9 |  | F25B5.2 | 5958725 | 5962230 |
| 9 |  | F25B5.1 | 5966362 | 5971430 |
| 9 |  | mlp-1 | 5971540 | 5973610 |
| 9 |  | frm-2 | 5976739 | 5982073 |
| 9 |  | T04C9.3 | 5985586 | 5985804 |
| 9 |  | T04C9.2 | 5991827 | 5992177 |
| 9 |  | T04C9.1 | 5993177 | 6006793 |
| 9 |  | ZK328.4 | 6006364 | 6009031 |
| 9 |  | eft-1 | 6009509 | 6012775 |
| 9 |  | npp-10 | 6013483 | 6018457 |
| 9 |  | cyk-3 | 6020112 | 6024585 |
| 9 |  | tbx-7 | 6025037 | 6026782 |
| 9 |  | ZK328.6 | 6028760 | 6030530 |
| 9 |  | ZK328.7 | 6032690 | 6039417 |
| 9 |  | F40H6.2 | 6040281 | 6044384 |
| 9 |  | tbx-11 | 6045718 | 6046852 |
| 9 |  | F40H6.5 | 6049262 | 6055394 |
| 9 |  | F40H6.t1 | 6056245 | 6056326 |
| 9 |  | F40H6.1 | 6057003 | 6058339 |
| 9 |  | F40H6.6 | 6065467 | 6066163 |
| 9 |  | C05D10.2 | 6067803 | 6072022 |
| 9 |  | C05D10.1 | 6080508 | 6084683 |
| 9 |  | C05D10.3 | 6084684 | 6087401 |
| 9 |  | C05D10.4 | 6088631 | 6102807 |
| 9 |  | T17E9.2 | 6114427 | 6116276 |
| 9 |  | kin-18 | 6118571 | 6124011 |
| 9 |  | Y42G9A.4 | 6124129 | 6136221 |
| 9 |  | Y42G9A.6 | 6142068 | 6149535 |
| 9 |  | Y42G9A.3 | 6146211 | 6148423 |
| 9 |  | Y42G9A.2 | 6148125 | 6148995 |
| 9 |  | Y42G9A.1 | 6149927 | 6155144 |
| 9 |  | F42A10.3 | 6154061 | 6157703 |
| 9 |  | F42A10.t1 | 6158723 | 6158804 |
| 9 |  | F42A10.t2 | 6158910 | 6158995 |
| 9 |  | nfm-1 | 6159787 | 6164518 |
| 9 |  | F42A10.9 | 6164592 | 6171122 |
| 9 |  | efk-1 | 6164845 | 6168499 |
| 9 |  | F42A10.5 | 6171092 | 6172747 |
| 9 |  | F42A10.1 | 6173250 | 6176732 |
| 9 |  | F42A10.6 | 6175542 | 6176808 |
| 9 |  | F42A10.7 | 6177875 | 6178633 |
| **9,8** |  |  | **6178994** | |
| 8 |  | nas-28 | 6181023 | 6183163 |
| 8 |  | C23G10.5 | 6191055 | 6192026 |
| 8 |  | C23G10.6 | 6192126 | 6194488 |
| 8 |  | C23G10.7 | 6195937 | 6198360 |
| 8 |  | rpn-2 | 6198846 | 6202539 |
| **8,7** |  |  | **6200925** | |
| 7 | *let-713 let-716 let-721 let-725 let-728 let-747 let-756 let-767 let-774 let-814 let-815 let-816 let-817 let-818* | rpn-2 | 6198846 | 6202539 |
| 7 |  | C23G10.8 | 6202571 | 6206147 |
| 7 |  | rps-3 | 6206745 | 6207910 |
| 7 |  | C23G10.2 | 6208133 | 6209087 |
| 7 |  | C23G10.1 | 6209228 | 6212771 |
| 7 |  | C23G10.10 | 6213056 | 6213867 |
| 7 |  | C23G10.11 | 6215109 | 6215486 |
| 7 |  | hot-4 | 6220498 | 6224316 |
| 7 |  | T12A2.15 | 6224435 | 6229347 |
| 7 |  | T12A2.5 | 6233662 | 6239280 |
| 7 |  | T12A2.3 | 6240772 | 6241524 |
| 7 |  | T12A2.6 | 6241794 | 6244327 |
| 7 |  | T12A2.7 | 6244640 | 6245772 |
| 7 |  | T12A2.8 | 6244797 | 6248178 |
| 7 |  | T12A2.2 | 6248261 | 6251551 |
| 7 |  | T12A2.1 | 6251756 | 6271161 |
| 7 |  | srg-8 | 6253497 | 6254781 |
| 7 |  | srg-9 | 6255403 | 6256719 |
| 7 |  | srg-1 | 6257154 | 6258306 |
| 7 |  | srg-2 | 6258534 | 6260159 |
| 7 |  | srg-4 | 6260160 | 6262029 |
| 7 |  | srg-5 | 6262453 | 6263704 |
| 7 |  | srg-6 | 6264404 | 6265955 |
| 7 |  | srg-7 | 6266454 | 6268103 |
| 7 |  | srg-3 | 6269025 | 6270321 |
| 7 |  | C18F10.9 | 6271505 | 6272903 |
| 7 |  | C18F10.2 | 6273104 | 6275960 |
| 7 |  | C18F10.7 | 6276006 | 6278301 |
| 7 |  | hmg-1.2 | 6280288 | 6282361 |
| 7 |  | F47D12.5 | 6282389 | 6285607 |
| 7 |  | F47D12.6 | 6286607 | 6287238 |
| 7 |  | F47D12.7 | 6293257 | 6295794 |
| 7 |  | F47D12.3 | 6298437 | 6299269 |
| 7 |  | F47D12.9 | 6299287 | 6303163 |
| 7 |  | gar-2 | 6309322 | 6320853 |
| 7 |  | F47D12.10 | 6321330 | 6321929 |
| 7 |  | C56G2.5 | 6322240 | 6324179 |
| 7 |  | C56G2.4 | 6331670 | 6335774 |
| 7 |  | let-767 | 6338464 | 6339914 |
| 7 |  | C56G2.7 | 6338479 | 6341870 |
| 7 |  | C56G2.9 | 6342476 | 6343232 |
| 7 |  | C56G2.3 | 6346921 | 6348001 |
| 7 |  | C56G2.1 | 6353329 | 6365409 |
| 7 |  | C56G2.15 | 6366565 | 6367764 |
| 7 |  | tag-213 | 6367984 | 6373844 |
| 7 |  | rps-13 | 6374041 | 6374945 |
| 7 |  | C16A3.11 | 6374365 | 6374491 |
| 7 |  | tag-182 | 6375321 | 6379113 |
| 7 |  | C16A3.6 | 6379391 | 6380598 |
| 7 |  | C16A3.5 | 6380637 | 6381458 |
| 7 |  | C16A3.4 | 6381574 | 6382966 |
| 7 |  | C16A3.3 | 6383494 | 6389343 |
| 7 |  | C16A3.2 | 6389775 | 6390591 |
| 7 |  | C16A3.1 | 6390886 | 6393782 |
| 7 |  | C16A3.10 | 6394186 | 6396124 |
| 7 |  | let-756 | 6407071 | 6409960 |
| 7 |  | C05D11.5 | 6410043 | 6411501 |
| 7 |  | nas-4 | 6411541 | 6414934 |
| 7 |  | C05D11.7 | 6415720 | 6421653 |
| 7 |  | C05D11.8 | 6422400 | 6427246 |
| 7 |  | C05D11.9 | 6427470 | 6430876 |
| 7 |  | C05D11.10 | 6430979 | 6431734 |
| 7 |  | tag-170 | 6432123 | 6433089 |
| 7 |  | vps-16 | 6433180 | 6436431 |
| 7 |  | C05D11.1 | 6436550 | 6439891 |
| 7 |  | mel-32 | 6440087 | 6442942 |
| 7 |  | C05D11.13 | 6443057 | 6443913 |
| 7 |  | let-721 | 6444644 | 6446885 |
| 7 |  | T26A5.6 | 6448017 | 6450647 |
| 7 |  | set-1 | 6450734 | 6452314 |
| 7 |  | T26A5.5 | 6454423 | 6459952 |
| 7 |  | T26A5.4 | 6460062 | 6462147 |
| 7 |  | T26A5.8 | 6462182 | 6462789 |
| 7 |  | dlc-1 | 6462847 | 6463743 |
| 7 |  | tag-99 | 6465109 | 6467125 |
| 7 |  | T26A5.2 | 6467324 | 6471569 |
| 7 |  | tag-200 | 6471711 | 6475146 |
| 7 |  | F23F12.9 | 6478900 | 6481166 |
| 7 |  | F23F12.8 | 6483158 | 6488815 |
| 7 |  | rpt-3 | 6489733 | 6491222 |
| 7 |  | srb-11 | 6491308 | 6492847 |
| 7 |  | srb-10 | 6493493 | 6494721 |
| 7 |  | F23F12.13 | 6495621 | 6498117 |
| 7 |  | sdz-15 | 6498584 | 6499963 |
| 7 |  | F23F12.3 | 6500399 | 6507657 |
| 7 |  | F23F12.12 | 6508576 | 6509388 |
| 7 |  | acr-5 | 6509639 | 6514705 |
| 7 |  | K03F8.1 | 6511209 | 6511721 |
| 7 |  | K03F8.t1 | 6513877 | 6513958 |
| 7 |  | C09E7.10 | 6518842 | 6519967 |
| 7 |  | oig-1 | 6524042 | 6525562 |
| 7 |  | pqn-10 | 6528165 | 6531962 |
| 7 |  | srxa-5 | 6532500 | 6533708 |
| 7 |  | C09E7.4 | 6534407 | 6536935 |
| 7 |  | C09E7.5 | 6538059 | 6541295 |
| 7 |  | C09E7.9 | 6541548 | 6545113 |
| 7 |  | C09E7.6 | 6545280 | 6546344 |
| 7 |  | C09E7.7 | 6546682 | 6551169 |
| 7 |  | C09E7.8 | 6553515 | 6558515 |
| 7 |  | F27B3.5 | 6559039 | 6562835 |
| **7,6** |  |  | **6559307** | **6562088** |
| 6 | *let-755 let-765* | F27B3.5 | 6559039 | 6562835 |
| 6 |  | F27B3.5 | 6559039 | 6562835 |
| 6 |  | F27B3.7 | 6562962 | 6563599 |
| 6 |  | F27B3.6 | 6577833 | 6578524 |
| 6 |  | F27B3.8 | 6579638 | 6579817 |
| 6 |  | acr-21 | 6583451 | 6585557 |
| 6 |  | F20H11.5 | 6587804 | 6589213 |
| 6 |  | F20H11.1 | 6588533 | 6592115 |
| 6 |  | nsh-1 | 6592710 | 6598470 |
| 6 |  | F20H11.6 | 6598655 | 6601083 |
| 6 |  | mdh-1 | 6606055 | 6608053 |
| 6 |  | F20H11.4 | 6608563 | 6610271 |
| 6 |  | srh-40 | 6610762 | 6612203 |
| 6 |  | Y40D12A.2 | 6612463 | 6615074 |
| 6 |  | Y40D12A.1 | 6615354 | 6619985 |
| **6,5** |  |  | **6620106** | |
| 5 | *let-711 let-714 let-717 let-724 let-764 let-769 let-771 let-783 let-792 let-793 let-794 let-795 let-843* | C13B9.2 | 6620847 | 6622134 |
| 5 |  | C13B9.3 | 6622616 | 6624912 |
| 5 |  | C13B9.1 | 6628946 | 6631480 |
| 5 |  | C13B9.4 | 6632781 | 6652023 |
| 5 |  | M01G4.1 | 6664900 | 6665314 |
| 5 |  | rbf-1 | 6675741 | 6703914 |
| 5 |  | isw-1 | 6703933 | 6709995 |
| 5 |  | F37A4.6 | 6705755 | 6707138 |
| 5 |  | F37A4.5 | 6711882 | 6713046 |
| 5 |  | F37A4.4 | 6713521 | 6717959 |
| 5 |  | F37A4.3 | 6718634 | 6719682 |
| 5 |  | bath-41 | 6720353 | 6722196 |
| 5 |  | F37A4.2 | 6722454 | 6723222 |
| 5 |  | F37A4.1 | 6722483 | 6725649 |
| 5 |  | F37A4.t1 | 6724073 | 6724158 |
| 5 |  | Y102E9.2 | 6725725 | 6727996 |
| 5 |  | Y102E9.t1 | 6726929 | 6727014 |
| 5 |  | Y102E9.5 | 6728093 | 6730108 |
| 5 |  | odr-4 | 6730719 | 6733870 |
| 5 |  | Y102E9.3 | 6733930 | 6734458 |
| 5 |  | Y102E9.6 | 6735906 | 6736580 |
| 5 |  | K07E12.2 | 6737410 | 6737685 |
| 5 |  | dig-1 | 6746248 | 6794701 |
| 5 |  | R05H11.1 | 6795576 | 6797659 |
| 5 |  | R05H11.2 | 6798077 | 6799924 |
| 5 |  | tag-204 | 6802725 | 6815715 |
| 5 |  | F28F5.t1 | 6805505 | 6805577 |
| 5 |  | F28F5.4 | 6816580 | 6816817 |
| 5 |  | F28F5.6 | 6817266 | 6819311 |
| 5 |  | ten-1 | 6828947 | 6847222 |
| 5 |  | R13F6.5 | 6847341 | 6848992 |
| 5 |  | srv-1 | 6851189 | 6853069 |
| 5 |  | zak-1 | 6852705 | 6856720 |
| 5 |  | R13F6.8 | 6858731 | 6859509 |
| 5 |  | R13F6.2 | 6859801 | 6860724 |
| 5 |  | sma-3 | 6860901 | 6863907 |
| 5 |  | R13F6.10 | 6865159 | 6869359 |
| 5 |  | kbp-1 | 6869576 | 6870236 |
| 5 |  | K04C2.8 | 6870498 | 6871063 |
| 5 |  | K04C2.3 | 6872986 | 6873668 |
| 5 |  | K04C2.7 | 6882193 | 6883621 |
| 5 |  | med-2 | 6884441 | 6884965 |
| 5 |  | brd-1 | 6885142 | 6889460 |
| 5 |  | K04C2.t3 | 6887666 | 6887738 |
| 5 |  | K04C2.t2 | 6888547 | 6888618 |
| 5 |  | K04C2.t1 | 6888743 | 6888814 |
| 5 |  | K04C2.2 | 6889711 | 6895026 |
| 5 |  | K04C2.5 | 6890981 | 6891541 |
| 5 |  | T07E3.4 | 6897952 | 6900533 |
| 5 |  | brc-2 | 6900523 | 6903043 |
| 5 |  | T07E3.3 | 6903832 | 6904971 |
| 5 |  | T07E3.2 | 6907539 | 6907904 |
| 5 |  | T07E3.6 | 6909375 | 6912418 |
| 5 |  | F57B9.7 | 6924047 | 6928751 |
| 5 |  | inf-1 | 6929975 | 6931889 |
| 5 |  | byn-1 | 6931935 | 6935798 |
| 5 |  | F57B9.8 | 6933613 | 6935265 |
| 5 |  | coq-2 | 6935903 | 6939329 |
| 5 |  | F57B9.3 | 6940113 | 6941672 |
| 5 |  | pqn-46 | 6941825 | 6944674 |
| 5 |  | let-711 | 6946701 | 6958814 |
| 5 |  | rpn-6 | 6959051 | 6961962 |
| 5 |  | F57B9.1 | 6962105 | 6963192 |
| 5 |  | F31E3.4 | 6963559 | 6968050 |
| 5 |  | eft-3 | 6969569 | 6971654 |
| 5 |  | rfc-4 | 6972146 | 6973591 |
| 5 |  | F31E3.2 | 6974279 | 6977958 |
| 5 |  | ceh-20 | 6979818 | 6981724 |
| 5 |  | F31E3.6 | 6982446 | 6982998 |
| 5 |  | prk-1 | 6990107 | 7002233 |
| 5 |  | C06E8.5 | 7002319 | 7005298 |
| 5 |  | F11H8.2 | 7009081 | 7019509 |
| 5 |  | col-8 | 7019529 | 7020535 |
| 5 |  | rfl-1 | 7021101 | 7023517 |
| 5 |  | cyk-1 | 7023576 | 7029130 |
| 5 |  | F11H8.t1 | 7030491 | 7030562 |
| 5 |  | R01H2.7 | 7035384 | 7037228 |
| 5 |  | R01H2.2 | 7056509 | 7058427 |
| 5 |  | egg-2 | 7058573 | 7060528 |
| 5 |  | R01H2.4 | 7061042 | 7064000 |
| 5 |  | R01H2.1 | 7062811 | 7063125 |
| 5 |  | ger-1 | 7065494 | 7067371 |
| 5 |  | ubc-18 | 7067642 | 7068501 |
| 5 |  | R01H2.8 | 7069034 | 7069491 |
| 5 |  | ZK418.3 | 7070212 | 7070816 |
| 5 |  | lin-37 | 7070806 | 7072485 |
| 5 |  | ZK418.5 | 7072659 | 7073776 |
| 5 |  | ZK418.6 | 7074215 | 7078765 |
| 5 |  | ZK418.7 | 7079007 | 7080718 |
| 5 |  | ZK418.8 | 7083676 | 7084990 |
| 5 |  | ZK418.9 | 7085130 | 7087670 |
| 5 |  | ZK418.10 | 7088573 | 7090061 |
| 5 |  | ZK418.2 | 7091234 | 7093327 |
| 5 |  | nhr-9 | 7095482 | 7097008 |
| 5 |  | ZK418.11 | 7100411 | 7101854 |
| 5 |  | cpg-2 | 7102021 | 7103919 |
| 5 |  | B0280.6 | 7105370 | 7106389 |
| 5 |  | odd-1 | 7108251 | 7109239 |
| 5 |  | B0280.7 | 7110249 | 7111802 |
| 5 |  | nhr-10 | 7112227 | 7115367 |
| 5 |  | B0280.9 | 7120836 | 7122824 |
| 5 |  | B0280.3 | 7123053 | 7124258 |
| 5 |  | B0280.2 | 7124616 | 7128211 |
| 5 |  | B0280.13 | 7128830 | 7131977 |
| 5 |  | B0280.10 | 7132064 | 7133607 |
| 5 |  | B0280.1 | 7133718 | 7136337 |
| 5 |  | B0280.17 | 7135013 | 7136056 |
| 5 |  | B0280.11 | 7136471 | 7138175 |
| 5 |  | glr-2 | 7138612 | 7144761 |
| 5 |  | sls-2.5 | 7140376 | 7140482 |
| 5 |  | sls-2.16 | 7141320 | 7141428 |
| 5 |  | sls-2.18 | 7142220 | 7142326 |
| 5 |  | ogt-1 | 7145556 | 7153699 |
| 5 |  | nuo-4 | 7155612 | 7158741 |
| 5 |  | rnp-7 | 7158844 | 7161086 |
| 5 |  | K04G7.11 | 7161085 | 7162044 |
| 5 |  | K04G7.1 | 7162641 | 7164864 |
| 5 |  | F37C12.7 | 7167767 | 7171266 |
| 5 |  | srb-8 | 7171786 | 7173519 |
| 5 |  | srb-9 | 7174002 | 7175735 |
| 5 |  | srb-7 | 7177459 | 7178782 |
| 5 |  | rps-14 | 7178439 | 7179533 |
| 5 |  | rpl-36 | 7180174 | 7180606 |
| 5 |  | F37C12.3 | 7180659 | 7181837 |
| 5 |  | F37C12.2 | 7181920 | 7183915 |
| 5 |  | F37C12.1 | 7184277 | 7187780 |
| 5 |  | F37C12.14 | 7184966 | 7185530 |
| 5 |  | F37C12.10 | 7187818 | 7188476 |
| 5 |  | rps-21 | 7189063 | 7189567 |
| 5 |  | mec-14 | 7190136 | 7192921 |
| 5 |  | F37C12.18 | 7194051 | 7195353 |
| 5 |  | F37C12.13 | 7196154 | 7197997 |
| 5 |  | pfd-5 | 7198081 | 7198813 |
| 5 |  | dpy-31 | 7202590 | 7207577 |
| 5 |  | rpl-6 | 7208361 | 7209791 |
| 5 |  | R151.11 | 7208542 | 7208673 |
| 5 |  | R151.12 | 7208948 | 7209078 |
| 5 |  | R151.13 | 7209329 | 7209459 |
| 5 |  | R151.2 | 7209896 | 7215202 |
| 5 |  | R151.1 | 7217768 | 7219545 |
| 5 |  | R151.4 | 7219903 | 7221115 |
| 5 |  | R151.6 | 7221670 | 7223058 |
| 5 |  | R151.7 | 7223162 | 7226638 |
| 5 |  | R151.8 | 7226732 | 7229716 |
| 5 |  | R151.10 | 7229984 | 7230870 |
| 5 |  | adr-2 | 7230893 | 7232813 |
| 5 |  | T20H4.5 | 7232855 | 7234131 |
| 5 |  | prs-1 | 7238951 | 7241213 |
| 5 |  | T20H4.2 | 7241319 | 7242829 |
| 5 |  | osm-10 | 7243234 | 7245640 |
| 5 |  | T21D11.1 | 7244892 | 7247713 |
| 5 |  | B0361.4 | 7258940 | 7268820 |
| 5 |  | psd-1 | 7268342 | 7275323 |
| 5 |  | B0361.6 | 7275432 | 7277201 |
| **5,4** |  |  | **7277442** | |
| 4 | *let-702* | B0361.11 | 7277531 | 7281102 |
| 4 |  | B0361.3 | 7281331 | 7283861 |
| 4 |  | B0361.7 | 7283026 | 7285810 |
| 4 |  | B0361.10 | 7285924 | 7287530 |
| 4 |  | B0361.8 | 7287792 | 7290491 |
| 4 |  | B0361.2 | 7293561 | 7308291 |
| 4 |  | B0361.9 | 7298294 | 7299570 |
| 4 |  | acbp-4 | 7308281 | 7308817 |
| 4 |  | F56C9.6 | 7308925 | 7310697 |
| 4 |  | F56C9.3 | 7311224 | 7313036 |
| 4 |  | F56C9.7 | 7313070 | 7314906 |
| 4 |  | F56C9.8 | 7316406 | 7318688 |
| 4 |  | F56C9.11 | 7324883 | 7329534 |
| 4 |  | F56C9.10 | 7329185 | 7335055 |
| 4 |  | gsp-2 | 7336513 | 7343559 |
| 4 |  | F56C9.12 | 7337819 | 7337929 |
| 4 |  | kap-1 | 7338928 | 7343573 |
| 4 |  | F08F8.6 | 7343698 | 7345751 |
| 4 |  | F08F8.7 | 7345788 | 7347078 |
| 4 |  | F08F8.8 | 7347187 | 7349091 |
| 4 |  | F08F8.9 | 7349219 | 7354016 |
| 4 |  | F08F8.10 | 7354118 | 7357312 |
| 4 |  | F08F8.4 | 7357588 | 7358996 |
| 4 |  | F08F8.2 | 7359423 | 7362026 |
| 4 |  | F08F8.5 | 7362544 | 7363389 |
| 4 |  | F08F8.1 | 7364532 | 7366332 |
| 4 |  | T20B12.5 | 7366642 | 7367037 |
| 4 |  | T20B12.4 | 7367635 | 7369463 |
| 4 |  | mml-1 | 7370056 | 7374650 |
| 4 |  | T20B12.7 | 7377779 | 7378972 |
| 4 |  | hmg-4 | 7379079 | 7381521 |
| 4 |  | T20B12.3 | 7382038 | 7383831 |
| 4 |  | tbp-1 | 7383956 | 7386057 |
| 4 |  | T20B12.1 | 7386552 | 7391061 |
| 4 |  | T20B12.9 | 7391610 | 7395208 |
| **4,3** |  |  | **7395219** | |
| 3 | *let-768* | alh-12 | 7453937 | 7455897 |
| 3 |  | Y69F12A.1 | 7456874 | 7458446 |
| 3 |  | Y69F12A.3 | 7458740 | 7458871 |
| 3 |  | H14A12.7 | 7459183 | 7459419 |
| 3 |  | mls-1 | 7460266 | 7461838 |
| 3 |  | H14A12.6 | 7462192 | 7462320 |
| 3 |  | H14A12.3 | 7463738 | 7465094 |
| 3 |  | fum-1 | 7465530 | 7467552 |
| 3 |  | H14A12.5 | 7466874 | 7468014 |
| **3,2B** |  |  | **7476402** | |
| 2B | *let-704 let-743 let-746 let-751 let-752 let-821 let-841 let-842 mup-4* | mup-4 | 7479530 | 7489000 |
| 2B |  | cgh-1 | 7495244 | 7497758 |
| 2B |  | C07H6.10 | 7496498 | 7496575 |
| 2B |  | C07H6.9 | 7498743 | 7499289 |
| 2B |  | C07H6.4 | 7499633 | 7503154 |
| 2B |  | C07H6.3 | 7508120 | 7515425 |
| 2B |  | clk-2 | 7515147 | 7518448 |
| 2B |  | cux-7 | 7518564 | 7520729 |
| 2B |  | C07H6.2 | 7520928 | 7521362 |
| 2B |  | lig-4 | 7521387 | 7524737 |
| 2B |  | lin-39 | 7528593 | 7536557 |
| 2B |  | mir-231 | 7544996 | 7545019 |
| 2B |  | ceh-13 | 7555584 | 7558132 |
| 2B |  | R13A5.4 | 7562095 | 7564452 |
| 2B |  | R13A5.6 | 7564478 | 7565268 |
| 2B |  | R13A5.3 | 7566495 | 7567351 |
| 2B |  | R13A5.7 | 7567390 | 7570056 |
| 2B |  | rpl-9 | 7571708 | 7572756 |
| 2B |  | lpd-7 | 7573271 | 7575188 |
| 2B |  | R13A5.9 | 7575201 | 7579334 |
| 2B |  | R13A5.10 | 7582035 | 7582902 |
| 2B |  | cup-5 | 7584129 | 7591488 |
| 2B |  | R13A5.11 | 7591516 | 7593265 |
| 2B |  | T04A6.3 | 7606784 | 7610591 |
| 2B |  | T04A6.2 | 7614790 | 7615555 |
| 2B |  | T04A6.1 | 7616456 | 7617518 |
| 2B |  | fbn-1 | 7625891 | 7641003 |
| 2B |  | ZK783.2 | 7640980 | 7643029 |
| 2B |  | ZK783.t1 | 7642086 | 7642157 |
| 2B |  | ZK783.5 | 7646104 | 7646914 |
| 2B |  | ZK783.3 | 7647080 | 7647999 |
| 2B |  | ZK783.6 | 7648716 | 7649933 |
| 2B |  | flt-1 | 7650495 | 7655960 |
| 2B |  | C18H2.1 | 7662356 | 7672081 |
| 2B |  | C18H2.2 | 7678948 | 7680785 |
| 2B |  | C18H2.3 | 7684298 | 7687085 |
| 2B |  | C18H2.5 | 7689284 | 7694172 |
| 2B |  | C18H2.4 | 7694618 | 7700260 |
| 2B |  | lin-13 | 7700266 | 7709400 |
| 2B |  | C03B8.5 | 7706787 | 7706936 |
| 2B |  | C03B8.3 | 7712380 | 7712874 |
| 2B |  | C03B8.2 | 7716094 | 7716374 |
| 2B |  | C03B8.1 | 7716695 | 7717228 |
| 2B |  | ZK112.3 | 7726100 | 7727786 |
| 2B |  | ZK112.4 | 7729637 | 7729939 |
| 2B |  | ncl-1 | 7731315 | 7737999 |
| 2B |  | ZK112.5 | 7738026 | 7739584 |
| 2B |  | ZK112.6 | 7739783 | 7740469 |
| 2B |  | cdh-3 | 7741254 | 7754376 |
| 2B |  | pcp-1 | 7758658 | 7761196 |
| 2B |  | tag-354 | 7761908 | 7763529 |
| 2B |  | ZK686.2 | 7763653 | 7765980 |
| 2B |  | ZK686.5 | 7766409 | 7767116 |
| 2B |  | ZK686.3 | 7767499 | 7768991 |
| 2B |  | ZK686.4 | 7769143 | 7772487 |
| 2B |  | ZK686.1 | 7773062 | 7773751 |
| 2B |  | bath-15 | 7774808 | 7775942 |
| 2B |  | mab-5 | 7776835 | 7783382 |
| 2B |  | C08C3.4 | 7793438 | 7795266 |
| 2B |  | egl-5 | 7814310 | 7817055 |
| 2B |  | egl-45 | 7824311 | 7828553 |
| 2B |  | ZK652.6 | 7829336 | 7832420 |
| 2B |  | ceh-23 | 7839521 | 7841418 |
| 2B |  | ZK652.8 | 7842308 | 7843563 |
| 2B |  | rpl-35 | 7855044 | 7856014 |
| 2B |  | tag-307 | 7855766 | 7857168 |
| 2B |  | coq-5 | 7857240 | 7858385 |
| 2B |  | tag-277 | 7858905 | 7859441 |
| 2B |  | tomm-7 | 7859539 | 7860085 |
| 2B |  | mir-356 | 7860098 | 7860118 |
| 2B |  | cuc-1 | 7860387 | 7860930 |
| 2B |  | snr-5 | 7861994 | 7862503 |
| 2B |  | C02C2.4 | 7862568 | 7864995 |
| 2B |  | cup-4 | 7866436 | 7868656 |
| 2B |  | tyr-1 | 7873706 | 7877025 |
| 2B |  | C02C2.5 | 7877174 | 7878767 |
| 2B |  | ZK688.9 | 7881101 | 7884882 |
| 2B |  | ZK688.11 | 7883709 | 7884892 |
| 2B |  | ZK688.5 | 7885019 | 7891544 |
| 2B |  | ZK688.3 | 7892280 | 7894554 |
| 2B |  | ZK688.4 | 7894953 | 7895199 |
| 2B |  | ZK688.10 | 7895751 | 7897897 |
| **2B, 2A** |  |  | **7896734** | |
| 2A |  | ZK688.10 | 7895751 | 7897897 |
| 2A |  | ZK688.2 | 7900960 | 7904402 |
| 2A |  | tag-282 | 7904886 | 7907772 |
| 2A |  | ZK688.1 | 7909573 | 7910687 |
| 2A |  | ZK688.7 | 7910781 | 7911549 |
| 2A |  | ZK688.12 | 7912846 | 7913488 |
| 2A |  | gly-3 | 7914245 | 7917698 |
| 2A |  | tag-250 | 7917923 | 7924796 |
| 2A |  | C29E4.14 | 7920069 | 7920929 |
| 2A |  | C29E4.13 | 7927315 | 7928663 |
| 2A |  | npp-15 | 7928979 | 7932922 |
| 2A |  | ran-2 | 7933554 | 7937631 |
| 2A |  | C29E4.10 | 7937786 | 7940676 |
| 2A |  | C29E4.12 | 7940989 | 7941530 |
| 2A |  | kle-2 | 7941646 | 7945513 |
| **2A,1B** |  |  | **7945392** | |
| 1B | *let-722 let-754 let-766* | kle-2 | 7941646 | 7945513 |
| 1B |  | C29E4.7 | 7945524 | 7946947 |
| 1B |  | col-90 | 7948767 | 7950889 |
| 1B |  | C29E4.8 | 7949253 | 7951844 |
| 1B |  | C29E4.11 | 7955778 | 7958034 |
| 1B |  | C29E4.9 | 7956022 | 7956575 |
| 1B |  | F54H12.4 | 7961525 | 7962670 |
| 1B |  | F54H12.2 | 7964510 | 7965769 |
| 1B |  | F54H12.5 | 7966571 | 7972031 |
| 1B |  | F54H12.7 | 7969290 | 7970162 |
| 1B |  | F54H12.6 | 7972335 | 7973315 |
| 1B |  | aco-2 | 7973646 | 7977254 |
| 1B |  | C06G4.4 | 7977179 | 7977622 |
| 1B |  | C06G4.t1 | 7978400 | 7978472 |
| 1B |  | clp-1 | 7980996 | 7986563 |
| 1B |  | C06G4.1 | 7987058 | 7990478 |
| 1B |  | C06G4.5 | 7990718 | 7996367 |
| 1B |  | C06G4.6 | 8004389 | 8005764 |
| 1B |  | cit-1.1 | 8005864 | 8009750 |
| 1B |  | cit-1.2 | 8010707 | 8013642 |
| 1B |  | F44B9.5 | 8013741 | 8016467 |
| 1B |  | lin-36 | 8016577 | 8020802 |
| 1B |  | F44B9.2 | 8022392 | 8025093 |
| **1B,1A** |  |  | **8023387** | |
| 1A | *let-709 let-712 let-729 let-740 let-750 let-786 let-834 let-835 let-838 let-839 let-844 let-972* | F44B9.2 | 8022392 | 8025093 |
| 1A |  | pqn-38 | 8025139 | 8027971 |
| 1A |  | F44B9.8 | 8028064 | 8029578 |
| 1A |  | F44B9.9 | 8029833 | 8030991 |
| 1A |  | F44B9.10 | 8031391 | 8033655 |
| 1A |  | dpf-6 | 8038169 | 8041302 |
| 1A |  | K12H4.6 | 8042505 | 8042802 |
| 1A |  | K12H4.5 | 8044408 | 8045130 |
| 1A |  | K12H4.4 | 8044935 | 8045860 |
| 1A |  | K12H4.3 | 8045961 | 8047530 |
| 1A |  | K12H4.2 | 8047640 | 8048307 |
| 1A |  | K12H4.7 | 8048351 | 8051497 |
| 1A |  | ceh-26 | 8067221 | 8071348 |
| 1A |  | K12H4.t1 | 8069332 | 8069403 |
| 1A |  | dcr-1 | 8071842 | 8080200 |
| 1A |  | apc-2 | 8080539 | 8083553 |
| 1A |  | K06H7.7 | 8083712 | 8084937 |
| 1A |  | grp-1 | 8085630 | 8088359 |
| 1A |  | K06H7.3 | 8088408 | 8091512 |
| 1A |  | idi-1 | 8091634 | 8092969 |
| 1A |  | K06H7.2 | 8093511 | 8096094 |
| 1A |  | K06H7.8 | 8096662 | 8098078 |
| 1A |  | K06H7.1 | 8099253 | 8100564 |
| 1A |  | plk-1 | 8101273 | 8104002 |
| 1A |  | C14B9.t1 | 8106579 | 8106651 |
| 1A |  | C14B9.3 | 8108716 | 8111101 |
| 1A |  | C14B9.t2 | 8109529 | 8109650 |
| 1A |  | gei-8 | 8111105 | 8125401 |
| 1A |  | rpl-21 | 8129447 | 8130159 |
| 1A |  | C14B9.10 | 8130788 | 8132606 |
| 1A |  | C14B9.2 | 8133589 | 8136574 |
| 1A |  | hsp-12.2 | 8138015 | 8140137 |
| 1A |  | C14B9.8 | 8138015 | 8144538 |
| 1A |  | D2007.4 | 8144940 | 8145899 |
| 1A |  | D2007.3 | 8148900 | 8149058 |
| 1A |  | D2007.2 | 8152394 | 8154436 |
| 1A |  | D2007.1 | 8155399 | 8155868 |
| 1A |  | D2007.5 | 8155916 | 8158171 |
| 1A |  | C50C3.7 | 8159156 | 8161264 |
| 1A |  | bath-42 | 8161374 | 8163311 |
| 1A |  | prp-8 | 8164303 | 8172615 |
| 1A |  | C50C3.12 | 8176410 | 8177169 |
| 1A |  | C50C3.5 | 8179181 | 8180222 |
| 1A |  | C50C3.2 | 8181858 | 8189296 |
| 1A |  | C50C3.1 | 8190729 | 8193586 |
| 1A |  | unc-36 | 8193776 | 8201127 |
| 1A |  | snb-5 | 8203011 | 8205034 |
| 1A |  | C30A5.6 | 8205257 | 8207283 |
| 1A |  | C30A5.10 | 8207379 | 8209766 |
| 1A |  | unc-86 | 8212686 | 8216546 |
| 1A |  | C30A5.4 | 8222146 | 8223712 |
| 1A |  | C30A5.3 | 8225016 | 8226292 |
| 1A |  | rfs-1 | 8226398 | 8232474 |
| 1A |  | C02F5.7 | 8229645 | 8232831 |
| 1A |  | C02F5.6 | 8232578 | 8235449 |
| 1A |  | C02F5.14 | 8234443 | 8235564 |
| 1A |  | C02F5.5 | 8236132 | 8236777 |
| 1A |  | tsp-2 | 8236904 | 8237793 |
| 1A |  | tsp-1 | 8237934 | 8239343 |
| 1A |  | C02F5.12 | 8240864 | 8242923 |
| 1A |  | pbs-6 | 8243039 | 8244014 |
| 1A |  | C02F5.4 | 8244363 | 8245653 |
| 1A |  | C02F5.3 | 8245811 | 8247168 |
| 1A |  | C02F5.13 | 8247271 | 8248573 |
| 1A |  | C02F5.10 | 8248711 | 8249392 |
| 1A |  | C02F5.2 | 8249557 | 8249907 |
| 1A |  | knl-1 | 8250556 | 8254242 |
| 1A |  | F09G8.5 | 8254895 | 8257505 |
| 1A |  | ncr-2 | 8258265 | 8265716 |
| 1A |  | col-91 | 8262297 | 8263585 |
| 1A |  | F09G8.3 | 8265982 | 8267739 |
| 1A |  | F09G8.7 | 8267867 | 8268274 |
| 1A |  | tag-198 | 8268566 | 8270453 |
| 1A |  | mps-4 | 8270710 | 8271315 |
| 1A |  | F09G8.10 | 8270710 | 8272088 |
| 1A |  | F09G8.8 | 8272895 | 8275891 |
| 1A |  | mig-10 | 8279028 | 8305734 |
| 1A |  | F10E9.7 | 8305390 | 8306275 |
| 1A |  | F10E9.5 | 8307512 | 8308329 |
| 1A |  | F10E9.4 | 8308470 | 8309594 |
| 1A |  | F10E9.3 | 8310924 | 8312491 |
| 1A |  | F10E9.2 | 8312638 | 8314205 |
| 1A |  | sas-4 | 8314306 | 8318500 |
| 1A |  | F10E9.11 | 8318665 | 8319057 |
| 1A |  | F10E9.10 | 8319399 | 8320199 |
| 1A |  | F10E9.1 | 8323106 | 8324299 |
| 1A |  | F10E9.12 | 8324646 | 8325465 |
| 1A |  | ZC262.3 | 8326499 | 8330866 |
| 1A |  | ZC262.8 | 8326525 | 8331838 |
| 1A |  | ZC262.2 | 8332142 | 8333366 |
| 1A |  | ZC262.4 | 8333582 | 8334152 |
| 1A |  | ZC262.1 | 8340845 | 8341329 |
| 1A |  | ZC262.5 | 8342143 | 8342529 |
| 1A |  | R05D3.5 | 8349040 | 8349524 |
| 1A |  | R05D3.6 | 8350338 | 8350724 |
| 1A |  | unc-116 | 8351168 | 8355093 |
| 1A |  | R05D3.8 | 8356838 | 8357727 |
| 1A |  | rfp-1 | 8358544 | 8362410 |
| 1A |  | R05D3.9 | 8362123 | 8368754 |
| 1A |  | R05D3.3 | 8364551 | 8366421 |
| 1A |  | R05D3.2 | 8372174 | 8375084 |
| 1A |  | met-2 | 8375219 | 8380752 |
| 1A |  | R05D3.12 | 8380864 | 8381460 |
| 1A |  | R05D3.1 | 8381635 | 8387418 |
| 1A |  | ZK353.4 | 8388426 | 8389558 |
| 1A |  | ZK353.10 | 8389908 | 8390590 |
| 1A |  | ZK353.5 | 8394129 | 8394957 |
| 1A |  | ZK353.3 | 8395904 | 8396646 |
| 1A |  | ZK353.2 | 8398643 | 8399055 |
| 1A |  | lap-1 | 8399123 | 8401849 |
| 1A |  | ZK353.7 | 8401968 | 8403526 |
| 1A |  | ubxn-4 | 8403651 | 8405481 |
| 1A |  | ZK353.1 | 8405793 | 8409321 |
| 1A |  | ZK353.9 | 8409415 | 8411935 |
| 1A |  | sor-1 | 8416146 | 8422568 |
| 1A |  | cec-1 | 8426256 | 8428500 |
| 1A |  | ZK1236.1 | 8429255 | 8431465 |
| 1A |  | ZK1236.5 | 8431519 | 8432131 |
| 1A |  | ZK1236.9 | 8432545 | 8433310 |
| 1A |  | ZK1236.8 | 8436617 | 8437728 |
| 1A |  | pqn-96 | 8438003 | 8439328 |
| 1A |  | ZK1236.7 | 8439385 | 8443526 |
| 1A |  | C30C11.4 | 8443825 | 8446828 |
| 1A |  | rpn-3 | 8447268 | 8449229 |
| 1A |  | C30C11.1 | 8449674 | 8450427 |
| 1A |  | F42H10.t1 | 8473386 | 8473458 |
| 1A |  | F42H10.5 | 8474560 | 8478290 |
| 1A |  | F42H10.6 | 8478319 | 8480368 |
| 1A |  | F42H10.3 | 8484071 | 8488118 |
| 1A |  | F42H10.7 | 8488278 | 8490260 |
| 1A |  | F42H10.2 | 8490558 | 8490961 |
| 1A |  | F42H10.9 | 8493791 | 8497256 |
| 1A |  | C04D8.1 | 8497541 | 8518794 |
| 1A |  | ZC21.8 | 8518871 | 8519856 |
| 1A |  | ZC21.9 | 8524879 | 8526125 |
| 1A |  | ZC21.3 | 8524989 | 8528525 |
| 1A |  | ZC21.10 | 8529992 | 8531034 |
| 1A |  | trp-1 | 8534996 | 8542646 |
| 1A |  | ZC21.6 | 8542863 | 8545519 |
| 1A |  | C02D5.3 | 8552496 | 8555590 |
| 1A |  | C02D5.2 | 8557244 | 8559103 |
| 1A |  | C02D5.1 | 8561094 | 8562881 |
| 1A |  | C06E1.3 | 8581011 | 8583818 |
| 1A |  | glr-1 | 8584064 | 8588607 |
| 1A |  | C06E1.5 | 8589244 | 8589396 |
| 1A |  | C06E1.6 | 8590741 | 8590890 |
| 1A |  | C06E1.7 | 8591179 | 8593000 |
| 1A |  | C06E1.8 | 8595544 | 8597751 |
| 1A |  | C06E1.1 | 8605740 | 8606412 |
| 1A |  | C06E1.9 | 8606412 | 8609691 |
| 1A |  | rha-2 | 8609972 | 8614464 |
| **1A,0** |  |  | **8611252** | |
| 0 |  | rha-2 | 8609972 | 8614464 |
| 0 |  | C06E1.11 | 8614670 | 8616118 |
| 0 |  | C13G5.2 | 8616752 | 8618347 |
| 0 |  | ceh-16 | 8622566 | 8625908 |
| 0 |  | polk-1 | 8626629 | 8628477 |
| 0 |  | gpr-1 | 8628843 | 8630685 |
| 0 |  | dnj-10 | 8631066 | 8633436 |
| 0 |  | twk-7 | 8633986 | 8643647 |
| 0 |  | F22B7.t1 | 8639214 | 8639333 |
| 0 |  | F22B7.4 | 8639699 | 8639936 |
| 0 |  | F22B7.t2 | 8646075 | 8646156 |
| 0 |  | F22B7.3 | 8648971 | 8649364 |
| 0 |  | F22B7.t5 | 8650289 | 8650360 |
| 0 |  | flp-23 | 8650639 | 8651992 |
| 0 |  | F22B7.9 | 8652482 | 8653814 |
| 0 |  | F22B7.t3 | 8652962 | 8653034 |
| 0 |  | F22B7.t4 | 8653515 | 8653587 |
| 0 |  | dpy-19 | 8659875 | 8667382 |
| 0 |  | F22B7.1 | 8665253 | 8666787 |
| 0 |  | pgl-2 | 8670456 | 8674958 |
| 0 |  | kin-31 | 8671611 | 8673533 |
| 0 |  | fli-1 | 8675230 | 8683982 |
| 0 |  | sup-5 | 8678404 | 8678475 |
| 0 |  | B0303.2 | 8684715 | 8686388 |
| 0 |  | B0303.3 | 8686180 | 8688364 |
| 0 |  | B0303.4 | 8689757 | 8693359 |
| 0 |  | B0303.7 | 8693457 | 8699362 |
| 0 |  | sdz-1 | 8699475 | 8700242 |
| 0 |  | B0303.9 | 8700836 | 8703213 |
| 0 |  | B0303.11 | 8703209 | 8710019 |
| 0 |  | B0303.16 | 8705018 | 8706254 |
| 0 |  | B0303.14 | 8713896 | 8716091 |
| 0 |  | B0303.15 | 8717549 | 8718406 |
| 0 |  | ZK370.3 | 8720108 | 8725883 |
| 0 |  | ZK370.4 | 8725239 | 8731280 |
| 0 |  | ZK370.5 | 8737592 | 8741321 |
| 0 |  | ZK370.6 | 8741643 | 8743747 |
| 0 |  | ZK370.7 | 8746187 | 8748609 |
| 0 |  | sma-2 | 8749679 | 8756976 |
| 0 |  | ZK370.8 | 8752008 | 8754658 |
| 0 |  | aak-1 | 8756914 | 8760997 |
| 0 |  | mig-22 | 8763198 | 8767249 |
| 0 |  | PAR2.1 | 8768216 | 8769146 |
| 0 |  | K02D10.1 | 8769417 | 8776047 |
| 0 |  | K02D10.2 | 8770663 | 8771132 |
| 0 |  | K02D10.3 | 8771241 | 8771858 |
| 0 |  | K02D10.5 | 8776160 | 8777802 |
| 0 |  | K02D10.4 | 8777815 | 8779919 |
| 0 |  | ztf-1 | 8787043 | 8792245 |
| 0 |  | zfp-1 | 8792754 | 8806181 |
| 0 |  | F54F2.6 | 8800393 | 8800608 |
| 0 |  | F54F2.7 | 8806180 | 8807253 |
| 0 |  | prx-19 | 8807388 | 8809236 |
| 0 |  | F54F2.1 | 8818809 | 8825177 |
| 0 |  | F54F2.9 | 8825150 | 8827134 |
| 0 |  | F44E2.3 | 8827318 | 8829194 |
| 0 |  | F44E2.10 | 8829598 | 8831016 |
| 0 |  | F44E2.4 | 8832397 | 8838331 |
| 0 |  | F44E2.6 | 8844479 | 8845092 |
| 0 |  | F44E2.7 | 8845781 | 8848393 |
| 0 |  | F44E2.8 | 8848540 | 8849776 |
| 0 |  | F44E2.9 | 8849898 | 8850439 |
| 0 |  | F44E2.2 | 8853710 | 8861133 |
| 0 |  | mir-80 | 8865192 | 8865214 |
| 0 |  | mir-227 | 8865235 | 8865256 |
| 0 |  | mir-238 | 8867250 | 8867272 |
| 0 |  | mir-90 | 8873909 | 8873930 |
| 0 |  | K01F9.2 | 8875266 | 8876932 |
| 0 |  | ZK637.1 | 8882876 | 8885392 |
| 0 |  | ZK637.2 | 8887657 | 8890056 |
| 0 |  | ZK637.14 | 8888276 | 8889013 |
| 0 |  | tag-256 | 8890138 | 8894113 |
| 0 |  | ZK637.4 | 8894120 | 8894563 |
| 0 |  | tag-205 | 8894993 | 8896552 |
| 0 |  | ZK637.6 | 8898891 | 8899436 |
| 0 |  | lin-9 | 8900144 | 8904040 |
| 0 |  | unc-32 | 8905525 | 8911649 |
| 0 |  | tpk-1 | 8912058 | 8913345 |
| 0 |  | trxr-2 | 8913677 | 8916354 |
| 0 |  | cdc-25.3 | 8916326 | 8917838 |
| 0 |  | ZK637.15 | 8920019 | 8921068 |
| 0 |  | ZK637.13 | 8922308 | 8923207 |
| 0 |  | ZK637.12 | 8924163 | 8925165 |
| 0 |  | ZK643.7 | 8925516 | 8926438 |
| 0 |  | ZK643.1 | 8934145 | 8935328 |
| 0 |  | ZK643.9 | 8937677 | 8937807 |
| 0 |  | ZK643.3 | 8944725 | 8953286 |
| 0 |  | ZK643.5 | 8952815 | 8956106 |
| 0 |  | ZK643.2 | 8956392 | 8957484 |
| 0 |  | ZK643.6 | 8957664 | 8958555 |
| 0 |  | grl-25 | 8962205 | 8965548 |
| 0 |  | R08D7.1 | 8965954 | 8967637 |
| 0 |  | R08D7.2 | 8967827 | 8969537 |
| 0 |  | eif-3.D | 8969556 | 8971488 |
| 0 |  | R08D7.4 | 8971802 | 8973569 |
| 0 |  | R08D7.8 | 8972616 | 8973581 |
| 0 |  | R08D7.5 | 8974096 | 8974836 |
| 0 |  | pde-2 | 8974977 | 8981808 |
| 0 |  | R08D7.7 | 8992136 | 8994738 |
| 0 |  | F59B2.2 | 8994589 | 8996697 |
| 0 |  | F59B2.3 | 8996801 | 8999696 |
